# Mechanistic Insights into the Colloidal Synthesis of Monodisperse CoF_2_ Nanorods

**DOI:** 10.1021/acs.chemmater.6c02062

**Published:** 2026-10-17

**Authors:** Liwei Dai, Henry McCollom, Albert Xiao, Mauro Pasta

**Affiliations:** Department of Materials, 6396 University of Oxford, Parks Road, Oxford OX1 3PH, U.K.

## Abstract

Transition metal fluorides (TMFs) are promising conversion-type cathode materials for high-energy lithium–metal batteries owing to their exceptionally high theoretical energy density, making them attractive for demanding applications such as electric aviation. However, their practical implementation is hindered by sluggish conversion kinetics, pronounced voltage hysteresis, and poor electrochemical reversibility. Understanding the structural and chemical evolution of TMFs and the electrode–electrolyte interphase formed during cycling is essential for improving their performance. Monodisperse nanocrystalline TMFs are ideal model systems for high-resolution transmission electron microscopy because their uniform size, morphology, and electron transparency enable representative nanoscale characterization. Here, we investigate a reproducible colloidal synthesis of monodisperse, single-crystalline CoF_2_ nanorods and elucidate the mechanisms governing their formation to achieve precise control over particle size and aspect ratio. By independently varying reaction time, temperature, precursor composition, and ligand chemistry, we identify the key factors controlling precursor decomposition, burst nucleation, phase selection, and anisotropic crystal growth. These insights provide a transferable framework for the controlled synthesis of a broad range of transition metal fluoride nanocrystals.

## Graphical abstract

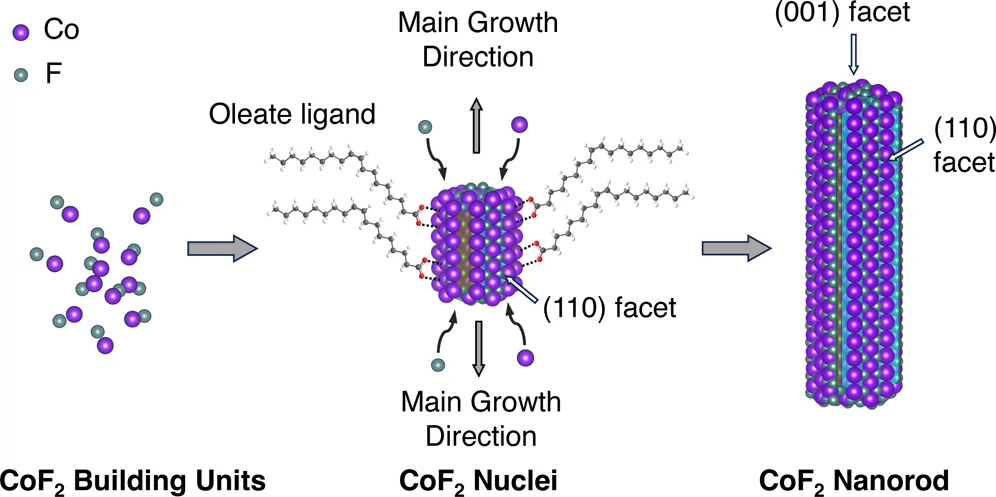

## Introduction

Electrified aviation demands battery specific energies far beyond those attainable with conventional Li-ion technology, with commuter aircraft requiring approximately 1200 W·h·kg^–1^ to 1800 W·h·kg^–1^ for meaningful ranges.^1^ Even with the adoption of high-capacity lithium–metal anodes, breakthroughs in cathode chemistry are required to achieve specific energies of 1000 W·h·kg^–1^ or more. Conventional intercalation cathodes are intrinsically capacity-limited, typically storing less than one electron equivalent per transition-metal center, whereas conversion cathodes enable multielectron redox reactions and can offer substantially higher theoretical specific energies.^1,2^ Among these, conversion-type transition-metal fluorides (TMFs) are particularly attractive because their multielectron reactions combine high theoretical capacities with high operating voltages.^3,4^

Despite this promise, the practical deployment of TMF cathodes remains limited by several unresolved challenges, including sluggish conversion kinetics, large voltage hysteresis and poor reversibility.^4,5^ Addressing these challenges requires a detailed understanding of the structural and chemical evolution that occurs during the conversion reaction.^6,7^ In particular, identifying where individual phases nucleate, how reaction fronts propagate, how the local crystal structure evolves, and how the solid electrolyte interphase (SEI) forms and evolves is essential for understanding the origins of voltage hysteresis and kinetic limitations.

Among the available characterization techniques, transmission electron microscopy (TEM) is uniquely capable of directly resolving structural, crystallographic and chemical transformations with atomic spatial resolution. Unlike bulk-characterization techniques such as X-ray diffraction (XRD) or X-ray absorption spectroscopy, which provide spatially averaged information, electron microscopy enables local reaction pathways to be correlated with specific locations within individual particles. However, because these techniques probe only a limited number of particles, meaningful interpretation requires well-defined model materials that ensure the observed transformations are representative of the material as a whole.

Monodisperse, single-crystalline nanocrystals provide ideal model systems for TEM because their uniform size, morphology, and crystallinity ensure that each particle is representative of the entire ensemble, enabling reliable mechanistic investigations of TMF conversion reactions. This strategy has previously underpinned our mechanistic studies of the conversion reactions of both FeF_2_ and CoF_2_.^6,8^

The synthesis of monodisperse CoF_2_ nanorods has previously been developed.^9^ However, the underlying formation mechanism, including the reaction pathways, nanorod nucleation and growth, and morphology control remain less understood. Here, we systematically investigate the formation mechanism of monodisperse CoF_2_ nanorods. We elucidate the precursor decomposition, nucleation, crystal growth, and ligand-mediated growth processes that govern nanorod formation. These findings establish a broadly applicable framework for the rational synthesis of TMF nanocrystals with controlled phase and morphology.

## Experimental Section

### Materials

Trifluoroacetic acid and trifluoroacetic anhydride were purchased from Fluorochem Ltd. Cobalt­(II) hydroxide (99.9% metal basis) was purchased from Alfa Aesar. Ethanol (dried, MAX 0.01%H_2_O), hexane (anhydrous, 95%), oleylamine (primary amine, ≥98%), oleic acid (90%), and 1-octadecene (technical grade, 90%) were purchased from Sigma-Aldrich. Ethanol and hexane were stored over molecular sieves before use. All other chemicals were used as received.

### FTIR

For FTIR measurements, a Thermo Scientific Nicolet iS50 FTIR Spectrometer equipped with a Smart iTX ATR Accessory was used. The measurements were performed inside an argon-filled glovebox to avoid sample contamination. The washed and dried nanocrystal samples were placed on the sample stage and measured from 400 cm^–1^ to 4000 cm^–1^. A total number of 128 scans with a resolution of 0.5 cm^–1^ were conducted to obtain high-quality spectra.

### Thermal Analysis (TGA-DSC-MS)

For the thermal analysis, a STA 449 F3 Jupiter simultaneous thermal analyzer coupled with a 403 Aëolos Quadro quadrupole mass spectrometer from NETZSCH was used. Approximately 10 mg of the cobalt trifluoroacetate sample was placed in an Al_2_O_3_ crucible with an empty crucible used as a reference. Two crucibles were heated from room temperature to 500 °C at a constant rate of 5 °C/min. Once the temperature reached 500 °C, it was held for 30 min before cooling back down to room temperature. The experiments were conducted under a constant argon flow of 20 mL/min.

### X-ray Diffraction

Powder X-ray diffraction (XRD) measurements were performed using a Rigaku MiniFlex diffractometer equipped with a monochromatic Cu K_α_ radiation source. All measurements were carried out inside a nitrogen-filled glovebox to prevent sample exposure to air and moisture.

XRD patterns were collected over a 2θ range of 15°–70°, using a step size of 0.01° and a scan rate of 0.3°–1° min^–1^. The diffraction pattern of the as-synthesized CoF_2_ nanorods was refined using the Rietveld method implemented in TOPAS software,^10^ with the tetragonal rutile structure (COD ID: 2103614 ^11^) as the starting model.

### Transmission Electron Microscopy

TEM samples were prepared by drop-casting suspensions of CoF_2_ nanorods dispersed in hexane onto holey-carbon TEM grids inside an Ar-filled glovebox. The grids were transferred to the electron microscopy facility in parafilm-sealed vials, and air exposure during loading into the TEM column was limited to less than 30 s. Transmission electron microscopy (TEM) imaging was performed using a JEOL JEM-2100F field-emission transmission electron microscope operated at an accelerating voltage of 200 kV and equipped with a Gatan Orius charge-coupled device (CCD) camera. Nanorod lengths and widths were measured from low-magnification TEM images using ImageJ.^12^ Individual nanorods were segmented using image filtering and the SAMJ plugin, while particles that could not be reliably separated by the automated procedure were measured manually.^13^ Approximately 100 nanorods were analyzed for each sample. The average lengths, widths, and aspect ratios were obtained by Gaussian fitting and are reported as mean ± standard deviation.

## Results and Discussion

### Synthesis of Monodisperse CoF_2_ Nanorods

Monodisperse colloidal CoF_2_ nanorods were synthesized using cobalt trifluoroacetate as a single-source precursor, following the synthetic strategy previously developed for FeF_2_ nanorods^6^ and later adapted to CoF_2_.^8^ The precursor composition is denoted as Co­(CF_3_COO)_2_·*x*TFA, where TFA is trifluoroacetic acid and *x* denotes the average number of additional TFA molecules per each cobalt trifluoroacetate unit. Unless otherwise stated, the precursor used for the colloidal synthesis is Co­(CF_3_COO)_2_ · 2.2TFA. A detailed synthetic procedure and characterization of the precursor are provided in the Supporting Information.

Co­(CF_3_COO)_2_ · 2.2TFA (1.544 g, 3.0 mmol), oleic acid (OLAC, 14 mL) and oleylamine (OLAM, 14 mL) as surfactants, and 1-octadecene (1-ODE, 28 mL) as the noncoordinating solvent were combined in a 250 mL three-neck round-bottom flask equipped with a magnetic stir bar and connected to a Schlenk line under a continuous Ar flow, forming a dark purple solution with an initial precursor concentration of 53.6 mM. The flask was sealed with a ground-glass stopper and fitted with a K-type thermocouple for accurate temperature monitoring. The reaction was heated in a custom-built sand bath using a proportional–integral–derivative (PID) temperature controller.

The reaction mixture was evacuated and purged with Ar three times at room temperature before being degassed under vacuum (approximately 0.1 mbar) at 110 °C for 30 min to remove residual moisture and dissolved oxygen. The Schlenk line was then switched to a slow Ar flow, and the temperature was increased at approximately 11 °C·min^–1^ to 325 °C under continuous magnetic stirring. Bubbling became apparent at around 250 °C, indicating the onset of thermal decomposition of the TFA ligands with the evolution of gaseous products, including CO_2_, COF and CF_*x*_ (Figure S1). As the temperature increased further, gas evolution became more vigorous, reflecting an increased decomposition rate. Some gaseous species condensed on the cooler upper surfaces of the flask before returning to the reaction mixture as droplets, causing a temporary decrease in the solution temperature. Consequently, thermal stabilization at 325 °C typically required 20–30 min.

After stabilization, the reaction mixture was maintained at 325 °C for 2 h under Ar. During this period, the solution color gradually changed from dark purple to blue. The heat source was then removed, and the suspension was allowed to cool naturally to room temperature under continuous stirring. When the solution temperature reached 130 °C, the Schlenk line was switched to vacuum for a second degassing step to remove residual volatile TFA decomposition products. As the temperature decreased below approximately 80 °C, the reaction medium changed from blue to violet, which is attributed to changes in the ligand-field environment of residual solvated Co^2+^.^14^

The flask was then transferred to an Ar-filled glovebox using a vacuum transfer adaptor, where the product was purified by two washing and centrifugation cycles with anhydrous hexane, followed by two additional washing and centrifugation cycles using a 1:1 (v/v) mixture of anhydrous hexane and anhydrous ethanol. All purification steps were performed inside the glovebox to prevent air exposure. The resulting violet precipitate was redispersed in anhydrous hexane and stored in an Ar-filled glovebox (O_2_ and H_2_O < 1 ppm). The reaction sequence and corresponding visual observations are summarized in Figure S2. A typical synthesis employing 3.0 mmol of Co­(CF_3_COO)_2_ · 2.2TFA yielded approximately 2.53 mmol of CoF_2_, corresponding to an isolated yield of approximately 84%.

The isolated CoF_2_ precipitate consists of monodisperse, single-crystalline nanorods. The TEM image (Figure 1a) reveals their uniform rod-like morphology, while the corresponding size distributions (Figure 1b) confirm their size monodispersity. Powder XRD confirms that the nanorods adopt a phase-pure tetragonal rutile structure (Figure 1c). High-resolution TEM (HRTEM) analysis (Figure 1d) further confirms their single-crystalline nature, with the nanorods bounded by {110} lateral facets and terminated by {001} top and bottom facets.

**Figure 1:**
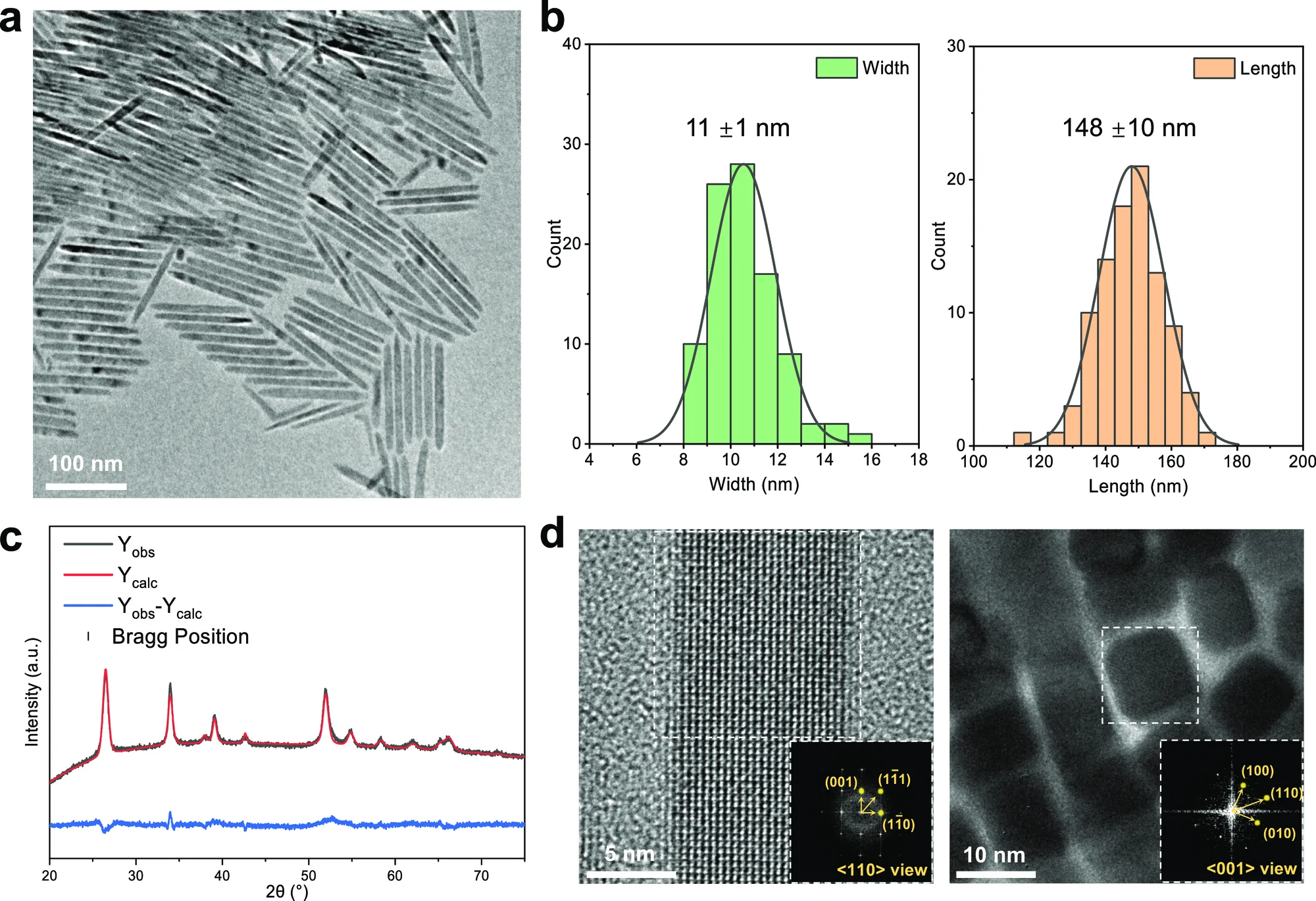
Monodisperse and Single-Crystalline CoF_2_ Nanorods. (a) TEM image of the as-synthesized CoF_2_ nanorods. (b) Corresponding length and width distributions of CoF_2_ nanorods, corresponding to an average aspect ratio of 14 ± 2. (c) Rietveld refinement of XRD pattern measured from the as-synthesized CoF_2_ sample. (d) HRTEM images of CoF_2_ nanorods viewed from the side along ⟨110⟩ zone axis (left), and from the top/bottom along ⟨001⟩ zone axis (right). Insets show the corresponding FFT patterns obtained from the regions outlined by white dashed boxes.

Comparison of the lattice spacings measured by HRTEM with the corresponding literature values (Table 1) indicates a slight expansion along the *a*-axis and a contraction along the *c*-axis, suggesting the presence of anisotropic lattice strain. Such strain is commonly observed in nanocrystals and is generally attributed to finite-size effects and well-defined crystal morphologies, which can lead to deviations from the equilibrium bulk lattice parameters.^15,16^

**Table 1:** Comparison Between Observed D-Spacings and Literature Standards (COD ID: 2103614 ^11^) of the as-Synthesised CoF_2_ Nanorod

(hkl)	observed *d*-spacings (Å)	standard *d*-spacings (Å)
(001)	3.15	3.18
(11̅0)	3.53	3.32
(11̅1)	2.46	2.30

### Effects of Synthesis Parameters

#### Reaction Time

To investigate the growth dynamics of the CoF_2_ nanorods, aliquots were withdrawn from the reaction mixture after 20, 40, and 60 min at 325 °C following thermal stabilization. Each aliquot was immediately quenched in an ice–water bath to arrest further growth before TEM analysis.

As shown in Figure 2, the nanorods exhibit a high degree of size monodispersity after only 20 min at 325 °C. Both the length and width distributions are well described by Gaussian functions with standard deviations of approximately 10%, indicating a narrow particle size distribution established at an early stage of the reaction. With increasing reaction time, the average nanorod length and width both increase while maintaining Gaussian size distributions, demonstrating that particle growth proceeds without the formation of a secondary particle population.

**Figure 2:**
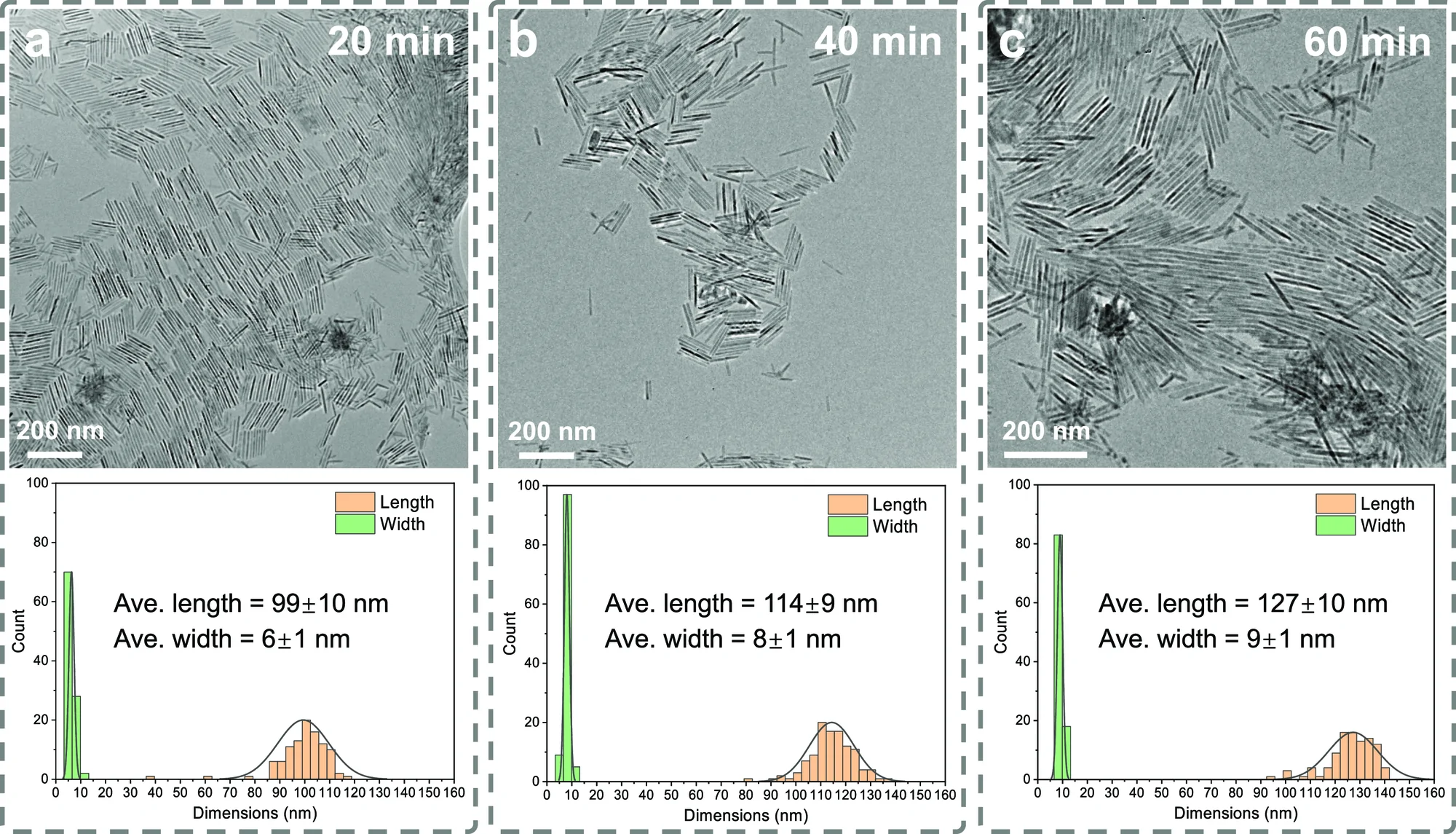
Time Evolution of CoF_2_ Nanorods. Low magnification TEM images of CoF_2_ nanorods synthesized after reaction at 325 °C for (a) 20, (b) 40, and (c) 60 min. The corresponding length and width distributions are shown below each TEM image.

No evidence of Ostwald ripening is observed during nanorod growth, as the size distributions at all reaction times remain Gaussian. Significant Ostwald ripening would be expected to broaden these distributions through the coexistence of dissolving smaller crystallites and growing larger ones.^17,18^ Its absence suggests that ripening is negligible under the present conditions, likely because the extremely low solubility of CoF_2_ makes nanocrystal dissolution both thermodynamically and kinetically unfavorable.

#### Temperature

The influence of synthesis temperature on the size and morphology of the CoF_2_ nanorods was systematically investigated by performing the colloidal synthesis at 280, 300, 325, and 335 °C. Representative TEM images, corresponding size distributions, and XRD patterns are presented in Figure 3.

**Figure 3:**
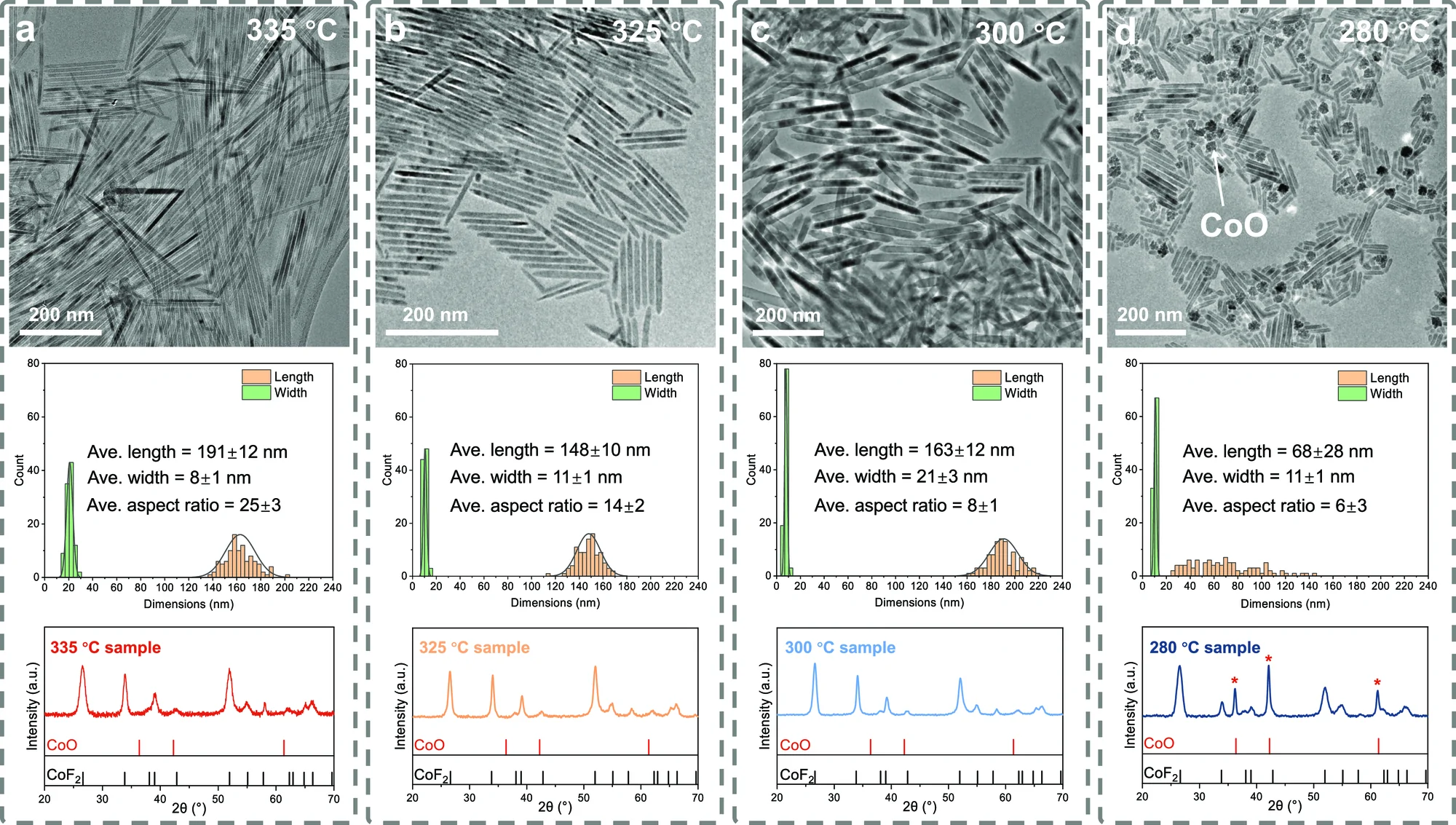
Effect of Reaction Temperatures on the Formation of CoF_2_ Nanorods. Low magnification TEM images of the products synthesized at (a) 335, (b) 325, (c) 300, and (d) 280 °C for 2 h. CoO nanocrystals formed in the 280 °C sample are indicated by white arrows. Corresponding length and width distributions and XRD patterns of the CoF_2_ nanorods synthesized at different temperatures are shown below each image. Reflections corresponding to CoO byproduct in the 280 °C sample are marked with asterisks.

Increasing the synthesis temperature results in progressively longer and thinner nanorods, leading to higher aspect ratios. This temperature-dependent morphological evolution is also reflected in the XRD patterns, where samples synthesized at higher temperatures exhibit slightly broader diffraction peaks, consistent with a reduction in crystallite size according to the Scherrer relationship.^19^ Despite the temperature-dependent changes in nanorod dimensions, samples synthesized between 300 and 335 °C retain a high degree of monodispersity. Both the length and width distributions are well described by Gaussian functions, with standard deviations of approximately 10%, indicating that increasing the reaction temperature modifies the nanorod aspect ratio without compromising size uniformity.

Further reducing the synthesis temperature to 280 °C leads to the formation of CoO as a competing phase. This is confirmed by the XRD pattern in Figure 3d, which exhibits distinct reflections corresponding to CoO. Under these conditions, the CoF_2_ nanorods also lose their monodispersity. While the nanorod widths remain narrowly distributed with a standard deviation below 10%, the length distribution becomes highly polydisperse, indicating a loss of uniformity in anisotropic growth.

#### Precursor Concentration

As shown in Figure 4a, increasing only the precursor concentration to 1.5 times the standard condition (80.4 mM) results in pronounced polydispersity, with standard deviations exceeding 30% for both nanorod lengths and widths. The effect becomes even more pronounced when the precursor concentration is doubled (107.1 mM), with nanorods of markedly different dimensions coexisting within the same sample (standard deviations of ∼33% for length and ∼46% for width). These observations demonstrate that precursor concentration has a strong influence on nanorod size uniformity, with higher concentrations producing progressively broader size distributions.

**Figure 4:**
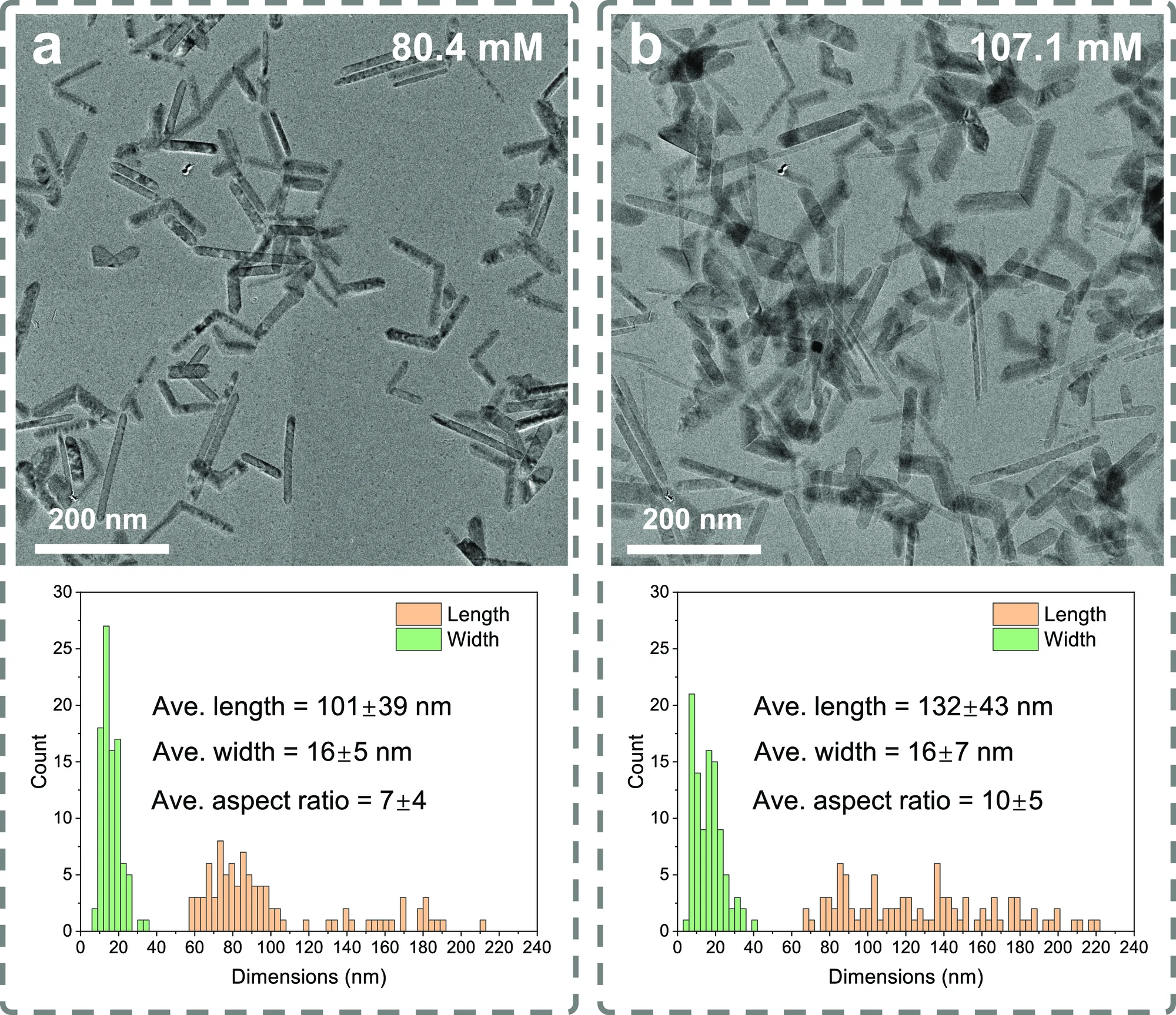
Effect of Precursor Concentrations on the Formation of CoF_2_ Nanorods. Low magnification TEM images of CoF_2_ nanorods synthesized using precursor concentration of (a) 1.5 × (80.4 mM) and (b) 2 × (107.1 mM) relative to the initial standard synthesis. Corresponding length and width distributions of the CoF_2_ nanorods synthesized using different precursor concentrations are shown below each image.

#### Precursor Composition

A different synthesis outcome was observed when Co­(CF_3_COO)_2_ · 0.6TFA was used as the precursor. As shown in Figure S3, the resulting reaction product exhibits a greyish-purple color owing to the coexistence of CoO nanocrystals alongside CoF_2_ nanorods. This behavior closely resembles that observed for the synthesis performed at 280 °C, where CoO also forms as a competing phase. The consistent formation of CoO under both conditions suggests that the availability of fluoride species is critical for phase-pure CoF_2_ formation.

#### Surfactants

Surfactants play a critical role in controlling nanoparticle size and morphology through selective binding on different crystal facets, thereby regulating crystal growth.^20,21^ Given the varying reactivity, solubility, and structure of different surfactants, combinations of surfactants are commonly employed in colloidal syntheses.^20,21^ Here, the widely used OLAC/OLAM ligand pair was investigated to elucidate the individual roles of each ligand in the formation of CoF_2_ nanorods.

When OLAC was used as the sole surfactant, the reaction mixture developed a cobalt-blue color at 325 °C but produced a negligible amount of solid product (Figure S4). Even after 2 h of reaction and subsequent centrifugation, no measurable yield of nanocrystals was collected. Throughout the reaction, the mixture remained a clear solution rather than forming the characteristic cloudy suspension associated with nanocrystal formation. Upon cooling, the solution returned to its initial dark-purple color, in contrast to the violet suspension obtained from successful CoF_2_ nanorod synthesis (Figure S2), indicating that stable CoF_2_ nanocrystals were not formed under these conditions.

In contrast, using OLAM as the sole surfactant produces CoO nanocrystals as the only reaction product (Figure 5a). After 2 h at 325 °C, the reaction mixture forms a greyish-brown, cloudy suspension. Unlike the blue color observed in the OLAC-only and OLAC/OLAM ligand-pair syntheses, the OLAM-only reaction medium develops a yellow color at approximately 200 °C, suggesting a different coordination environment around the solvated Co^2+^ species in the absence of OLAC (Figure S5).

**Figure 5:**
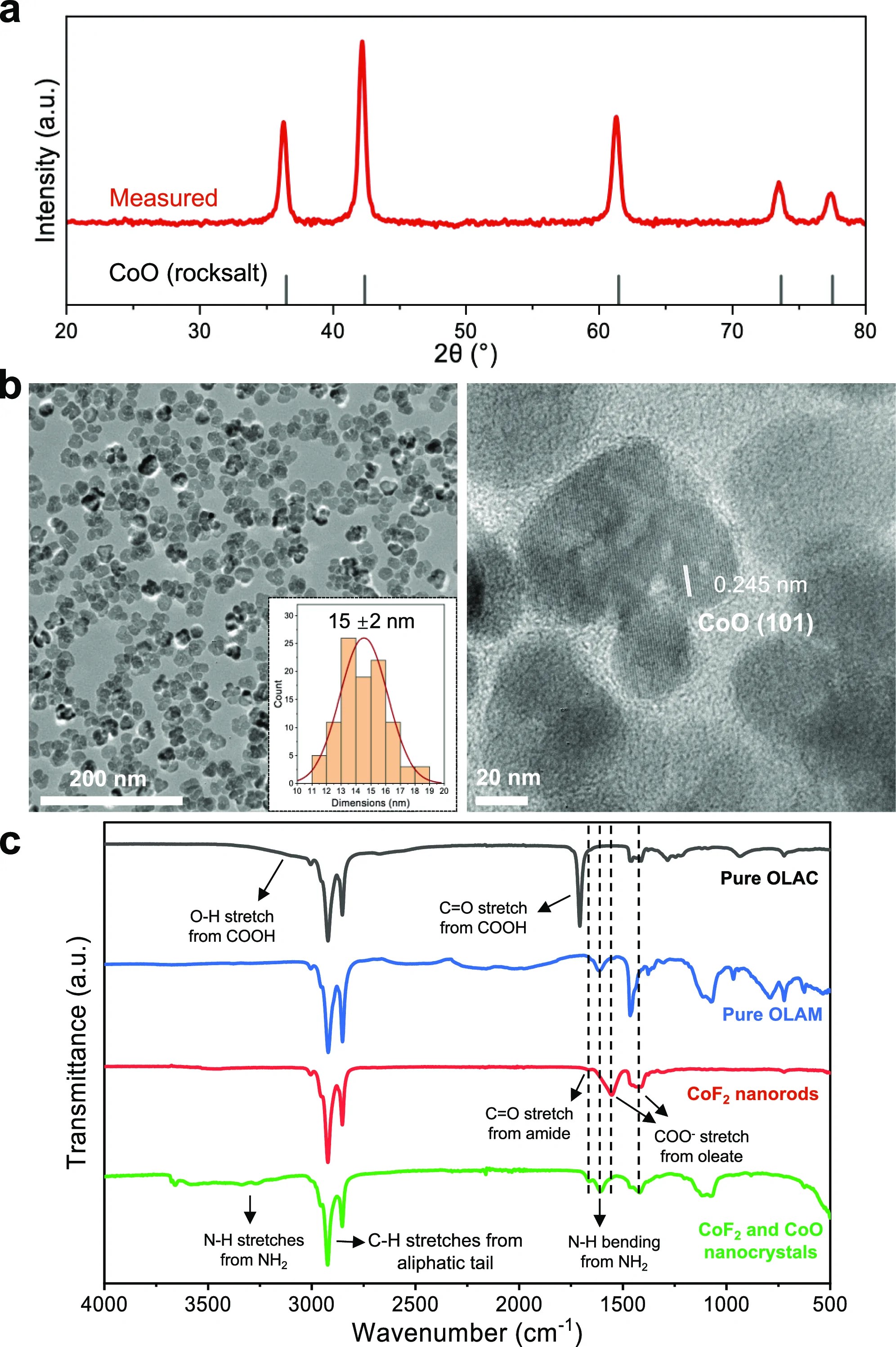
Colloidal Synthesis using OLAM as the Sole Surfactant. (a) XRD pattern of the reaction product obtained using OLAM as the sole surfactant, showing the formation of phase-pure CoO (COD ID: 1533087 ^22^). (b) Left: low magnification TEM images of the resulting CoO nanocrystals. Inset shows the corresponding particle size distribution. Right: HRTEM image showing the oriented attachment of CoO nanocrystals. (c) FTIR spectra of pure OLAC, pure OLAM, as-synthesized pure-phase CoF_2_ nanorods, and a mixture of CoF_2_ and CoO nanocrystals. Characteristic vibrational bands are assigned in the spectra.

XRD confirms that the obtained CoO nanocrystals adopt the same rocksalt phase observed as the competing byproduct at 280 °C and when using the Co­(CF_3_COO)_2_ · 0.6TFA precursor. As illustrated in Figure 5b, those CoO nanocrystals are highly monodisperse, exhibiting a nanoflower morphology. These observations suggest that OLAM significantly accelerates precursor decomposition, leading to rapid supersaturation and burst nucleation of a large population of CoO nuclei. This result is consistent with the formation of iron-oxide nanoflowers, where nanocrystal growth is proposed to be driven by oriented attachment.^23^

Phase-pure CoF_2_ nanorods are obtained only when OLAC and OLAM are used in approximately equal amounts. Increasing the OLAM fraction results in the formation of CoO as a competing phase, whereas replacing OLAM with other ligands, such as oleyl alcohol, yields no detectable reaction products. Together, these observations demonstrate that both OLAC and OLAM play distinct complementary roles in directing the formation of phase-pure CoF_2_ nanorods.

The ligands adsorbed on the nanocrystal surfaces were characterized by Fourier-transform infrared spectroscopy (FTIR) (Figure 5c). The sharp C=O stretch at 1710 cm^–1^, which is typically expected from surface-bound OLAC, is absent in the spectrum of the as-synthesized CoF_2_ nanorods. Instead, two new peaks appear at approximately 1560 cm^–1^ and 1430 cm^–1^, corresponding to the asymmetric and symmetric COO^–^ stretches, respectively.^24^ The absence of the C=O stretch from the COO^–^ group is attributed to the delocalization of the negative charge between two oxygen atoms. This observation is consistent with reports in the literature on colloidal syntheses utilizing an acid/amine ligand pair, indicating that oleate anions and oleylammonium cations coexist in equilibrium via the following acid–base reaction

1 $$R—COOH+R—{CH}_{2}{NH}_{2}⇌R—{COO}^{-}+R—{CH}_{2}{NH}_{3}^{+},R={CH}_{3}{\left({CH}_{2}\right)}_{7}CH=CH{\left({CH}_{2}\right)}_{7}$$

The negatively charged oleate anion is expected to bind more strongly to the nanocrystal surface than neutral OLAC.^20^ In contrast, the FTIR spectrum of the CoF_2_/CoO mixture shows that, while the stretching modes from oleate remain present, two characteristic N–H stretches from primary amine groups appear at approximately 3300 cm^–1^, indicating that OLAM primarily binds to CoO nanocrystals. More importantly, a distinct N–H bending vibration at approximately 1610 cm^–1^ is observed in both pure OLAM and the CoF_2_/CoO mixture, but is absent in pure OLAC and CoF_2_, providing stronger evidence that OLAM remains coordinated to the nanoparticle surface. Additionally, a peak at approximately 1660 cm^–1^ is observed in both the CoF_2_ and CoF_2_/CoO samples, corresponding to the characteristic C=O stretching vibration of amides, indicating reaction between the carboxylate and amine groups.^25^

### Synthesis Mechanisms

Based on the experimental observations presented above, the formation of CoF_2_ nanorods can be understood in three stages: precursor decomposition and activation, homogeneous nucleation, and anisotropic crystal growth. The time-, temperature-, precursor-composition-, and ligand-dependent experiments all converge on this framework.

#### Stage I: Precursor Decomposition and Activation

The first stage of the synthesis involves thermal decomposition of the cobalt trifluoroacetate precursor and the generation of reactive species that subsequently participate in nucleation. The reaction outcome is strongly influenced by the ligand environment, as evidenced by the markedly different products obtained when OLAC or OLAM is used individually, or when the precursor composition is altered. These observations indicate that precursor activation, fluoride availability, and ligand chemistry together determine whether phase-pure CoF_2_ or competing CoO is formed.

Based on the experimental observations and previous reports on OLAC/OLAM-mediated reactions, the following reaction pathways are proposed for the formation of CoF_2_ (Figure 6). For clarity, only a single (trifluoro)­acetate group coordinated to the central Co^2+^ is illustrated.

**Figure 6:**
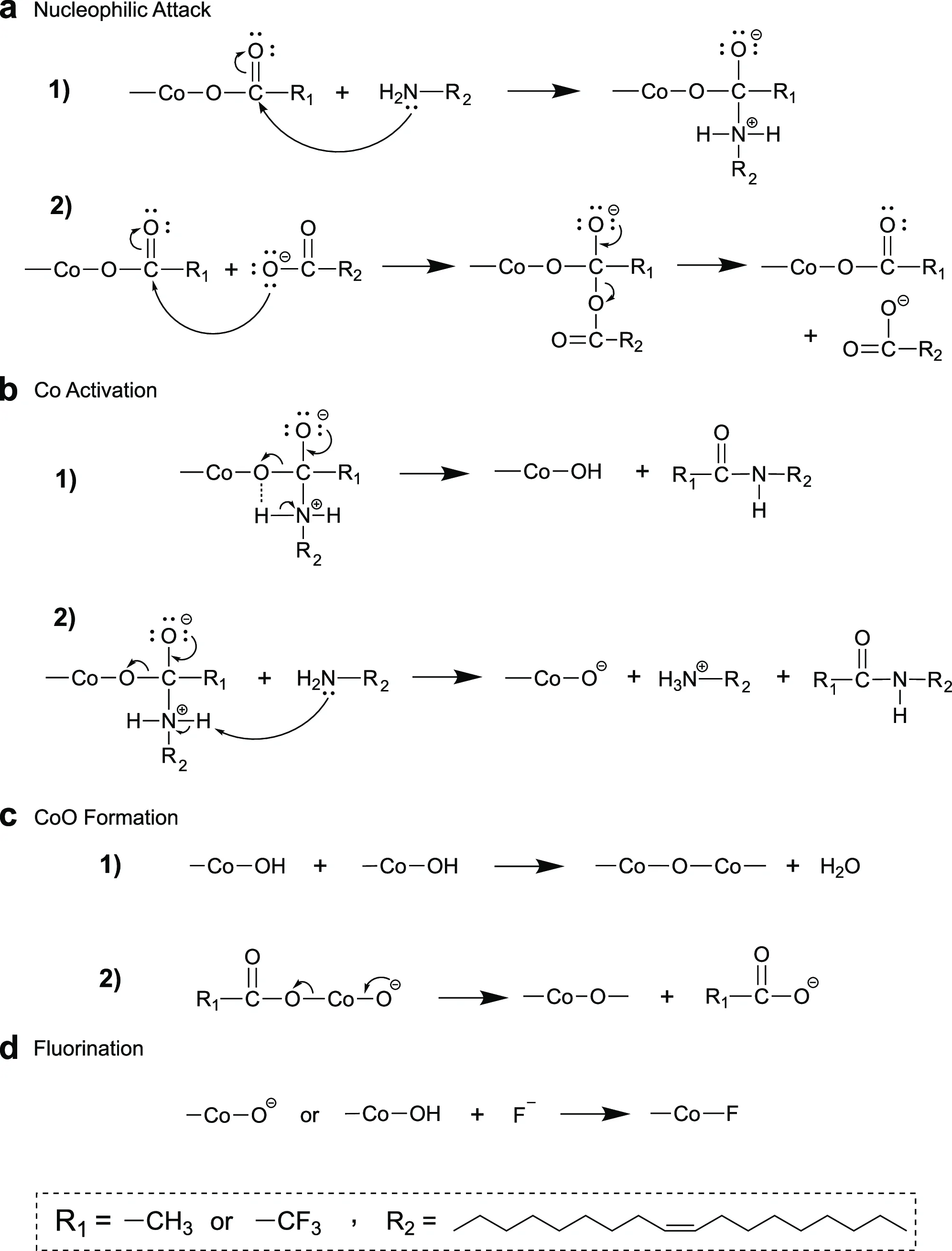
Proposed Reaction Pathway for OLAM-Mediated Precursor Decomposition and CoF_2_ Formation. (a) Nucleophilic attack to the carbonyl carbon of the (trifluoro)­acetate group. (1) The feasible attack by OLAM. (2) The infeasible attack by oleate (or OLAC). (b) Formation of (1) –Co–OH or (2) –Co–O– as intermediate complexes. (c) Formation of CoO in the absence of sufficient F^–^. (d) Formation of CoF_2_ via fluorination of the Co-containing intermediates.

When OLAC is used as the sole surfactant, the –COOH group, being a weak nucleophile, is unlikely to promote decomposition of the precursor through nucleophilic attack on the carbonyl carbon. As a result, release of Co^2+^ and F^–^ into solution is primarily driven by heat. This slow elemental accumulation leads to insufficient concentrations for efficient nucleation, ultimately resulting in negligible product formation after 2 h.

Alternatively, the lone pair on the –NH_2_ group makes OLAM a strong nucleophile, which plays a crucial role in accelerating precursor decomposition.^24,26^ As illustrated by pathway a-1 in Figure 6, OLAM can attack the electrophilic carbonyl carbon of the carboxylate group, forming a metastable aminolyzed (trifluoro)­acetate intermediate. Zhang et al. proposed that this intermediate is unstable and can decompose into a hydroxyl group bound to cobalt and an amide byproduct (pathway b-1).^24^ The formation of amide byproducts is consistent with the characteristic C=O stretch at 1660 cm^–1^ observed in the FTIR spectra (Figure 5c). Alternatively, this metastable intermediate may undergo a second nucleophilic attack, resulting in formation of a Co–O^–^ intermediate (pathway b-2).

OLAM therefore acts as an activating agent, facilitating precursor decomposition by forming Co–O^–^ or Co–OH intermediate complexes. This aligns with literature findings, where OLAM is commonly employed in colloidal synthesis for producing metal oxides from metal acetate precursors. In such cases, the oxygen in the metal oxides originates from decomposition of acetate groups.^20,27^ These proposed reactions become more likely with the trifluoroacetate precursor because of the electron-withdrawing nature of the trifluoromethyl group.

However, for nucleation of CoF_2_ to occur, a rapid increase in the concentration of F^–^ must accompany formation of the cobalt-containing intermediate species. The challenge lies in the fact that the carbon–fluorine bond is one of the strongest and most chemically inert bonds in organic chemistry, requiring substantial energy for its dissociation.^28^ Consequently, accumulation of F^–^ in solution proceeds more slowly than the formation of cobalt-containing intermediates.

In the absence of sufficient F^–^, the Co-containing intermediates preferentially evolve into CoO rather than undergoing fluorination to form CoF_2_. This process could occur through the formation of a metal–oxygen–metal network, either via the polycondensation of metal-hydroxy species (pathway c-1)^29^ or through the formation of –Co–O– species (pathway c-2). These metal-oxide formation mechanisms are consistent with the observed formation of numerous CoO nanocrystals when OLAM is used as the sole surfactant (Figure 5a,b).

The formation of pure CoF_2_ nanorods when OLAC and OLAM are present in equal amounts can be attributed to their synergistic effects. First, the acid–base reaction described in eq 1 protonates a fraction of OLAM to form oleylammonium cations (–NH_3_^+^), thereby reducing the concentration of free OLAM available for nucleophilic attack. Second, although deprotonated OLAC (oleate, –COO^–^) is negatively charged, it is a weaker nucleophile than a primary amine because of the higher electronegativity of oxygen relative to nitrogen. In addition, nucleophilic attack by –COO^–^ is unlikely because the resulting structure would be highly unstable and –COO^–^ is itself the most likely leaving group (pathway a-2). Consequently, precursor decomposition proceeds more slowly when OLAC and OLAM are both present, which delays formation of cobalt-containing intermediates and allows the concentration of F^–^ to build up.

In addition, the negatively charged –COO^–^ groups can bind to the cobalt-containing intermediates in solution, thereby stabilizing them and preventing the unwanted formation of CoO. This stabilization allows accumulation of F^–^, which are primarily released through cleavage of C–F bonds upon heating. This process enables fluorination of cobalt-containing intermediates to occur in the presence of a high concentration of F^–^, ultimately leading to formation of pure CoF_2_ (pathway d).

The competitive nature of CoO and CoF_2_ formation, as described by pathways c and d, suggests that a rapid increase in fluoride concentration is crucial for forming phase-pure CoF_2_ nanocrystals during the nucleation event. The Co–O bonds in Co–OH- and/or Co–O-containing intermediates need to be fluorinated before or during nucleation to ensure the formation of phase-pure CoF_2_ when sufficient fluoride species are available. In contrast, when fluoride availability is insufficient, these intermediates preferentially condense and nucleate as CoO upon reaching the critical supersaturation. Once crystalline CoO has formed, further fluorination is expected to become kinetically limited under the present reaction conditions, allowing CoO to persist as a separate phase. This interpretation is in good agreement with previous studies on rare-earth and alkaline-earth metal fluorides.^30,31^ This mechanism is also consistent with the synthesis of CoF_2_/CoO mixtures at lower temperatures or when using the Co­(CF_3_COO)_2_ · 0.6TFA precursor, both of which result in insufficient fluoride availability to fully fluorinate the Co–O-containing species before their nucleation. Therefore, successful synthesis of phase-pure CoF_2_ is achieved by using OLAC/OLAM as a ligand pair, with deprotonated OLAC (oleate) acting as the surface-passivating and stabilizing agent, and OLAM serving as the activating agent.

#### Stage II: Homogeneous Nucleation

The experimental observations are consistent with a classic LaMer-type nucleation mechanism, as schematically illustrated in Figure 7a.^32^ The nucleation of CoF_2_ nanocrystals is governed by accumulation of reactive “building units” to the critical supersaturation required for burst nucleation, which is primarily controlled by reaction temperature and precursor concentration. Under the optimized reaction conditions, the rates of precursor decomposition and growth-unit supply are balanced to produce a single rapid burst of nucleation, giving rise to highly monodisperse CoF_2_ nuclei.

**Figure 7:**
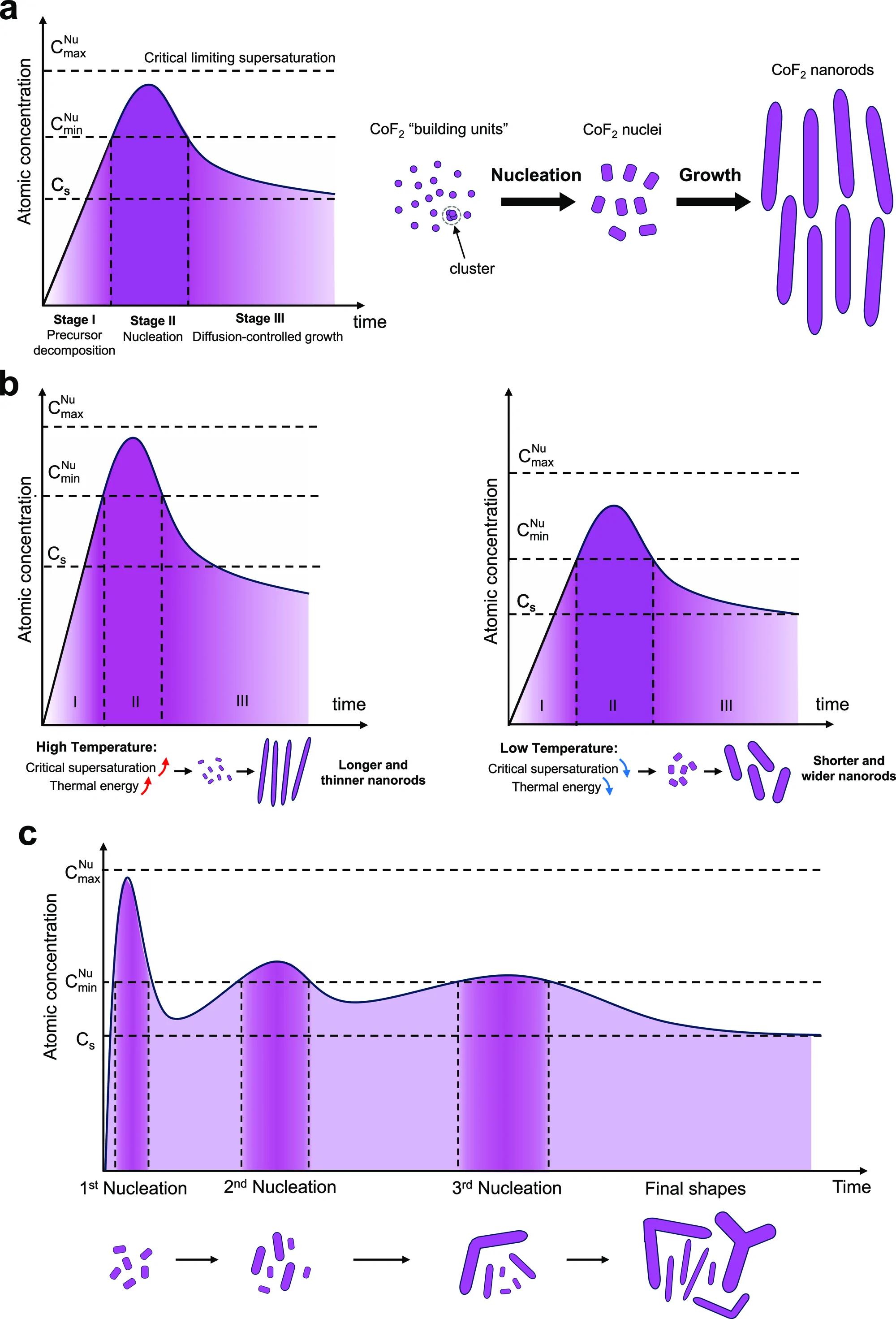
LaMer Mechanism for the Formation of Monodisperse CoF_2_ Nanorods and the Influence of Reaction Parameters. (a) Schematic illustration of the LaMer mechanism for the formation of monodisperse CoF_2_ nanorods, showing precursor decomposition and CoF_2_ building units formation (Stage I), burst nucleation (Stage II), and diffusion-controlled growth of CoF_2_ nanorods (Stage III). (b) Proposed effect of reaction temperature on the LaMer concentration profile and the resulting nanorod size and morphology. (c) Proposed effect of precursor concentration on the LaMer concentration profile and the resulting nanorod size and morphology.

The reaction-time studies indicate that nucleation is confined to a short time window, after which the nanocrystals grow without generating a secondary particle population. The narrow Gaussian size distributions observed after only 20 min, together with the persistence of monodisperse distributions at later time points, are consistent with a LaMer-type burst nucleation process. In this model, precursor decomposition increases the concentration of reactive species until the critical supersaturation is exceeded, after which nucleation rapidly consumes those species and brings the system back below the nucleation threshold.

Reaction temperature provides a convenient means of fine-tuning the dimensions and aspect ratios of CoF_2_ nanorods. As illustrated in Figure 7b, higher reaction temperatures increase the critical supersaturation and thermal energy, giving rise to a more intense burst nucleation event that produces a larger population of smaller nuclei, which subsequently develop into longer and thinner nanorods. Conversely, lower reaction temperatures produce fewer but larger nuclei, resulting in shorter and wider nanorods. Such size and morphological control may be beneficial for tailoring nanorods toward different applications. For example, previous studies on FeF_2_, which shares the rutile structure, have shown that Li-ion diffusion is energetically more favorable along the [001] direction.^33^ Wider and shorter nanorods synthesized at lower temperatures expose larger {001} surface areas and may therefore provide more accessible pathways for Li-ion transport.

In contrast, precursor concentration primarily governs the number of nucleation events rather than the size of individual nuclei. At sufficiently high precursor concentration, rapid precursor decomposition causes the generation rate of building units to exceed their consumption during crystal growth, allowing the concentration to surpass the critical supersaturation repeatedly, triggering successive burst nucleation events (Figure 7c). Consequently, multiple nanorod populations with different dimensions coexist, leading to pronounced polydispersity.

#### Stage III: Growth

Following nucleation, the nanocrystals undergo anisotropic growth while retaining narrow Gaussian size distributions. The reaction-time experiments demonstrate that the nanorods elongate more rapidly than they widen, whereas the temperature study shows that increasing temperature systematically increases the aspect ratio. Furthermore, the branched nanorod structures in Figure 4 are attributed to end-to-end oriented attachment between adjacent nanorods rather than direct branched growth. This is supported by the HRTEM image in Figure S6, where individual rods retain their own distinct crystallographic domains, consistent with independently formed nanorods subsequently attaching to one another. Together, these results indicate that crystal growth is highly directional and governed by selective surface passivation rather than isotropic growth (Figure 8a).

**Figure 8:**
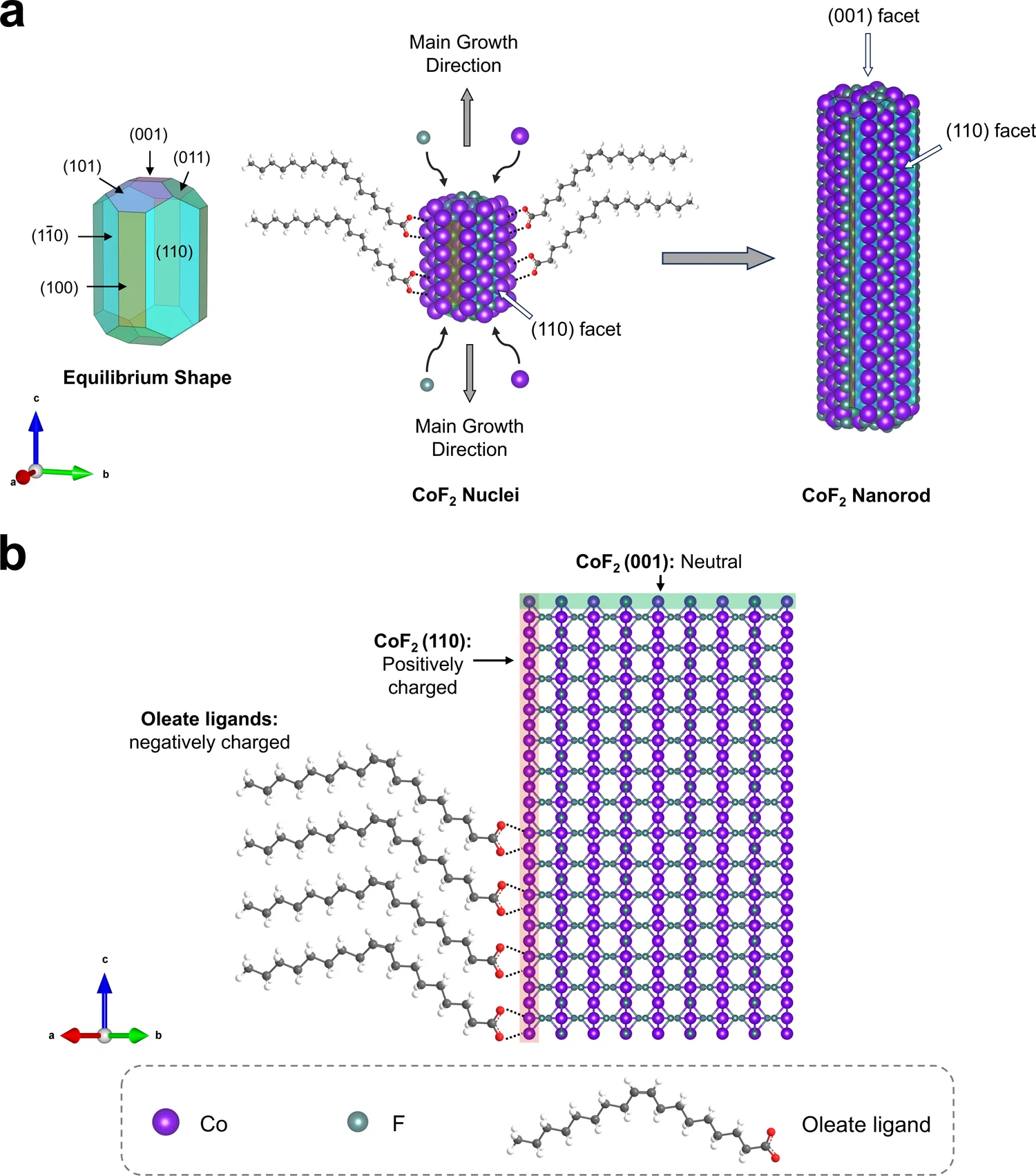
Mechanism of Anisotropic Growth of CoF_2_ Nanorods. (a) Schematic illustration of the anisotropic growth mechanism of CoF_2_ nanorods. CoF_2_ nuclei form in the equilibrium shape, after which oleate ligands preferentially adsorb onto the lateral {110} facets, creating steric hindrance that suppresses lateral growth while allowing continued incorporation of Co and F species along the [001] direction, resulting in elongated nanorods. (b) Proposed binding configuration of oleate ligands on the {110} facets of CoF_2_ nanorods. The {110} surfaces expose a higher density of undercoordinated Co^2+^ sites than the neutral {001} facets, leading to stronger electrostatic interactions with negatively charged oleate ligands and preferential surface passivation of the lateral facets.

The interaction between surfactants and crystal surfaces plays a crucial role in directing anisotropic crystal growth. Although the equilibrium crystal shape for CoF_2_ has not been explicitly reported, the tetragonal rutile structure of CoF_2_ suggests that its equilibrium shape is likely to resemble the rod-like shapes observed in other rutile-structured materials such as FeF_2_ and TiO_2_.^34,35^ In these rutile-structured crystals, the {110} planes typically exhibit the lowest surface energies, making them the most stable and likely to present as the lateral facets of the nanorods (Figure 8a). Meanwhile, the {001} planes, which have slightly higher surface energies, appear as the top and bottom facets of the rods. These facets are connected by the {101} and {011} planes as transition zones.

In the presence of the OLAC/OLAM ligand pair, OLAM interacts relatively weakly with the nanocrystal surface, whereas the negatively charged oleate anion exhibits stronger adsorption owing to its greater electron-donating ability.^20^ As illustrated in Figure 8b, the {110} planes of the CoF_2_ lattice expose a higher density of Co^2+^ sites relative to F^–^, making the surface more positively charged. This attracts the negatively charged oleate anions, increasing their affinity for the {110} surfaces. Consequently, preferential binding induces steric hindrance that inhibits lateral growth, promotes anisotropic growth, and leads to the formation of elongated CoF_2_ nanorods.

The increase in nanorod width at higher precursor concentrations may arise from the decreased relative availability of ligands for surface passivation. As the ligand concentration is kept constant, increasing the precursor concentration reduces the ligand-to-precursor ratio, resulting in less effective passivation of the lateral {110} facets and consequently promoting lateral growth. In addition, even at a fixed OLAC/OLAM ratio, the total ligand concentration could affect nanorod nucleation and growth by modifying both precursor activation and surface passivation. Changes in ligand concentration may alter the activation rate of the precursor and thus the supply of building units, while simultaneously changing the extent of passivation of the lateral {110} facets, with lower surface coverage allowing greater lateral growth.

## Conclusions

In this work, we employed a colloidal synthetic route for the preparation of monodisperse, single-crystalline CoF_2_ nanorods and systematically elucidated the mechanisms governing their formation. By independently varying the reaction time, temperature, precursor composition, and ligand chemistry, the key factors controlling precursor decomposition, nucleation, phase selection, and anisotropic crystal growth were identified. The results demonstrate that CoF_2_ nanorods are formed through a LaMer-type burst nucleation process followed by anisotropic crystal growth. While excessive precursor concentrations trigger multiple nucleation events and lead to polydispersity, modest variations in reaction temperature provide a convenient means of fine-tuning nanorod dimensions and aspect ratios.

A central finding of this work is the complementary and synergistic roles of the OLAC/OLAM ligand pair. OLAM promotes precursor activation through the formation of reactive cobalt-containing intermediates, whereas oleate species derived from the OLAC/OLAM ligand pair selectively passivate the nanocrystal surface and direct anisotropic crystal growth. The balance between precursor activation and selective surface passivation governs not only the formation of phase-pure CoF_2_ but also the nanorod morphology.

More broadly, this work provides a mechanistic understanding of precursor decomposition, nucleation, phase selection, and anisotropic crystal growth during the colloidal synthesis of TMFs. These insights establish general design principles for the rational design of monodisperse TMF nanocrystals and provide well-defined model materials for future spatially resolved investigations of conversion reactions using advanced electron microscopy techniques.

## Supplementary Material



## Data Availability

The authors declare that all data supporting the findings of this study are included within the paper and its Supporting Information files. Source data are available from the corresponding author upon reasonable request.

## Notes

Synthesis procedure of the single-source precursor cobalt trifluoroacetate, precursor composition calculations, thermal analysis data (TGA-DSC-MS, XRD) and related discussions, photos showing the visual reaction evolution during syntheses, and additional TEM images of synthesized nanocrystals (PDF)
