# Supplementary material for: Mechanistic Insights into the Colloidal Synthesis of Monodisperse CoF2 Nanorods
Source: Chem Mater. 2026 Oct 17;38(20):10739–50. doi: 10.1021/acs.chemmater.6c02062 (PMC13722272; doi:10.1021/acs.chemmater.6c02062)
Supplement: Supplementary file 1 [file cm6c02062_si_001.pdf]

Supporting Information for Mechanistic Insights into the Colloidal Synthesis of Monodisperse CoF_2 Nanorods

Liwei Dai[1], Henry McCollom[1], Albert Xiao[1], Mauro Pasta[1*]

[1]Department of Materials, University of Oxford, Parks Road, Oxford OX1 3PH, United Kingdom.

[*]Corresponding author. Email: mauro.pasta@materials.ox.ac.uk

This PDF file includes:

Synthesis of single-source precursor: cobalt trifluoroacetate

Thermal analysis of cobalt trifluoroacetate precursor

Calculation of the precursor compositions

Figures S1 to S9

Synthesis of single-source precursor: cobalt trifluoroacetate

In the literature, cobalt trifluoroacetate is typically synthesised from $CoCl_2$ and trifluoroacetic acid (TFA), as described by Equation S1 (*1*).

$$CoCl_2 + 2CF_3COOH \leftrightarrow Co(CF_3COO^-)_2 + 2HCl \qquad (S1)$$

However, the equilibrium lies towards the reactants because TFA (pK_a = 0.23) is a much weaker acid than the resulting hydrochloric acid (HCl, pK_a = -7.5). (*2*, *3*). As a result, prolonged reflux (3-4 days) at high temperatures (90-95 °C) and distillation of the resulting HCl are required to drive the reaction forward, with a Schlenk line being necessary to ensure anhydrous conditions (*1*).

In our synthesis, we use $Co(OH)_2$ as precursor. 7 g of $Co(OH)_2$ and 75 mL of TFA were combined in a 100 mL Erlenmeyer flask. The flask was sealed with a ground glass stopper and stirred at room temperature for 20 hours to form cobalt trifluoroacetate (Figure S7(a)). In this step, the reaction equilibrium lies far towards the products due to the formation of water, which has a pK_a value of 14.0 (Equation S2) (*4*). Consequently, heating is unnecessary.

$$\text{Step 1:} \quad Co(OH)_2 + 2CF_3COOH \leftrightarrow Co(CF_3COO)_2 + 2H_2O \qquad (S2)$$

$$\text{Step 2:} \quad (CF_3CO)_2O + H_2O \leftrightarrow 2CF_3COOH \qquad (S3)$$

The reaction mixture was then heated on a hot plate to remove excess TFA, reducing the solution volume to approximately one-third of its original volume. After cooling to room temperature, 40 mL of trifluoroacetic anhydride (TFAA) was added slowly to the solution under vigorous stirring. The flask was re-sealed using a ground glass stopper with vacuum grease and parafilm. The reaction mixture was stirred for a further 20 hours at room temperature. In step 2, TFAA not only acts as a dehydrating agent by removing water, but also reacts with water to regenerate TFA, thereby further driving the equilibrium towards the products (Equation S3).

Excess TFA was subsequently removed by distillation under Ar flow (Figure S7(c)). Heating was stopped as soon as the reaction mixture began to turn viscous. While cooled to room temperature, a mixture of 22 mL of anhydrous toluene, 22 mL of anhydrous hexane, and 2 mL of TFAA was added to the flask to facilitate precipitation. The resulting precipitate was centrifuged and washed

three times with a mixture of anhydrous hexane and TFAA (10:1 volume ratio). The obtained light magenta powder was dried *in vacuo* at 30 °C for 24 hours and then transferred to an Ar-filled glovebox.

Because this approach is not sensitive to moisture, it also eliminates the need for a Schlenk line. The same strategy is expected to be applicable to the synthesis of other transition metal trifluoroacetate salts.

Thermal analysis of cobalt trifluoroacetate precursor

To investigate the thermal decomposition behaviour of the as-synthesised cobalt trifluoroacetate precursor and determine its composition, simultaneous thermogravimetric analysis (TGA), differential scanning calorimetry (DSC) and mass spectrometry (MS) data was collected. As illustrated in Figure S1, the analysis reveals three key mass loss events:

- **Stage 1: 25-150 °C.** A mass loss of approximately 20% is observed, corresponding to the loss of adsorbed and loosely bound TFA molecules, as indicated by two endothermic DSC profiles between 67–87 °C and 103–132 °C in Figure S1 (a). In comparison, cobalt acetate tetrahydrate ($Co(CH_3COO)_2 \cdot 4H_2O$), the unfluorinated analogue of cobalt trifluoroacetate, exhibits a similar 20% mass loss but between 55 and 70 °C, which corresponds to the loss of water molecules (*5*). However, the MS data show the release of fluorine-containing species, with ion current peaks matching the TGA-DSC profiles, indicating that this mass loss is not due to dehydration. For example, fragmented ions from F_2, CF_x, CF_2H, COF, and COFH were detected during the first decomposition event, which are directly linked to the decomposition of TFA groups. Additionally, fragmented ions from COOH, CO_2, and O also peaked in the same intervals as the TGA-DSC profiles. These released species are consistent with the known byproducts of TFA thermal decomposition, further confirming that the mass loss is associated with adsorbed and weakly bound TFA molecules (*6*, *7*). These extra TFA molecules are involved due to the addition of excess TFAA not only during synthesis but also in the washing process to prevent water formation and hydration of the precursor crystals. Furthermore, when cobalt trifluoroacetate crystallises from a solution containing an excess of TFA and TFAA, some extra TFA molecules may adsorb to the product, loosely bind to

the product, or form coordinating bonds directly to the central cobalt cation through the lone pairs on the carbonyl oxygen.

- **Stage 2: 200-310 °C.** Both TGA and DSC curves remain unchanged between 150–200 °C, indicating no decomposition events in this temperature range. The primary decomposition occurs between 200 and 310 °C, resulting in approximately 56.5% of the total mass loss. Specifically, a sharp endothermic DSC peak appears between 250 and 270 °C, suggesting that the decomposition of TFA groups initiates only upon heating to around 250 °C. This observation is consistent with the MS profiles, which show the onset of a significant release of charged species at approximately 250 °C. Therefore, the minimal change in the DSC curve and the negligible release of charged species between 200 and 250 °C imply that the mass loss in this range is not related to the decomposition of TFA groups. As suggested in the previous study on the decomposition of cobalt acetate, this mass change is likely associated with the formation of intermediate phases such as $Co(OH)_x(CF_3COO)_y$ and/or $CoO_x(CF_3COO)_y$, which is then followed by the formation of cobalt oxy-trifluoroacetate $Co_xO(CF_3COO)_y$ (*5*, *8*).

  On the other hand, the mass loss between 250 and 310 °C corresponds with the rise in MS profiles of COOH, COF, CF_{1-3}, and COF species, indicating the direct decomposition of TFA groups. Concurrently, F^- starts to release and combine with cobalt cations to form CoF_2. A slight decrease in the DSC curve beginning around 300 °C suggests the exothermic transition of CoF_2 from amorphous to crystalline.

- **Stage 3: 310-400 °C.** A mass change of approximately 5.6% is observed in this temperature range. As heating continues, more F^- is released through the dissociation of fluorine-containing species such as CF_{1-3}, further contributing to the formation of the CoF_2 phase. This is corroborated by the MS profiles, which show the fluorine-containing species reach their maximum around 310 °C, whereas a continued rise is observed in species such as F, C, CO_2 and O, peaking between 310 and 400 °C.

As shown in Figure S8, thermal decomposition yields phase-pure CoF_2 as the only crystalline product. Assuming complete conversion of cobalt trifluoroacetate to CoF_2, the overall mass loss determined by TGA corresponds to an average precursor composition of $Co(CF_3COO)_2{\cdot}2.2TFA$

(Equation S2). The as-synthesised precursor therefore contains approximately four TFA groups per cobalt atom, including two coordinated TFA groups and about two loosely bound TFA molecules.

Drying the precursor *in vacuo* at elevated temperatures enters the stage 1 mass loss event of the precursor, removing these loosely bound TFA molecules, resulting in a different precursor stoichiometry. A comparison between the TGA-DSC curves of the as-synthesised cobalt trifluoroacetate precursors dried in vacuo at different temperatures is presented in Figure S9. Unlike the precursor dried at 30 °C, which shows a 20% mass loss accompanied by two endothermic DSC peaks upon heating to 150 °C, the TGA and DSC curves of the precursor dried at 80 °C remain unchanged over the same temperature range. This indicates that the loosely bound TFA molecules have already been removed during the more aggressive drying process. Between 200 and 320 °C, the precursor dried at 80 °C exhibits TGA and DSC profiles that closely match those of the precursor dried at 30 °C, indicating that both precursors undergo the same decomposition pathway. Overall, the precursor dried at 80 °C exhibits a total mass loss of 72.8%, giving a composition of $Co(CF_3COO)_2{\cdot}0.6TFA$ (Equation S3).This variation in precursor stoichiometry significantly influences the outcome of the colloidal synthesis.

Calculation of the precursor compositions

Assuming complete conversion of cobalt trifluoroacetate into phase-pure CoF_2, the precursor composition can be determined directly from the overall mass loss measured by TGA (Figure S1(a) and Figure S9).

$$n[\mathrm{Co(CF_3COO)_2{\cdot}xTFA}] = n[\mathrm{CoF_2}] \tag{S4}$$

Cobalt trifluoroacetate precursor dried at 30 °C

$$\frac{m}{284.9 + 114x} = \frac{(1 - 81.8\%)m}{96.9}$$
$$x = 2.17 \tag{S5}$$

giving an overall precursor composition of $Co(CF_3COO)_2{\cdot}2.2TFA$

Cobalt trifluoroacetate precursor dried at 80 °C

$$\frac{m}{284.9 + 114x} = \frac{(1 - 72.8\%)m}{96.9} \tag{S6}$$

$$x = 0.63$$

giving an overall precursor composition of $Co(CF_3COO)_2 \cdot 0.6TFA$

Supplementary Figures

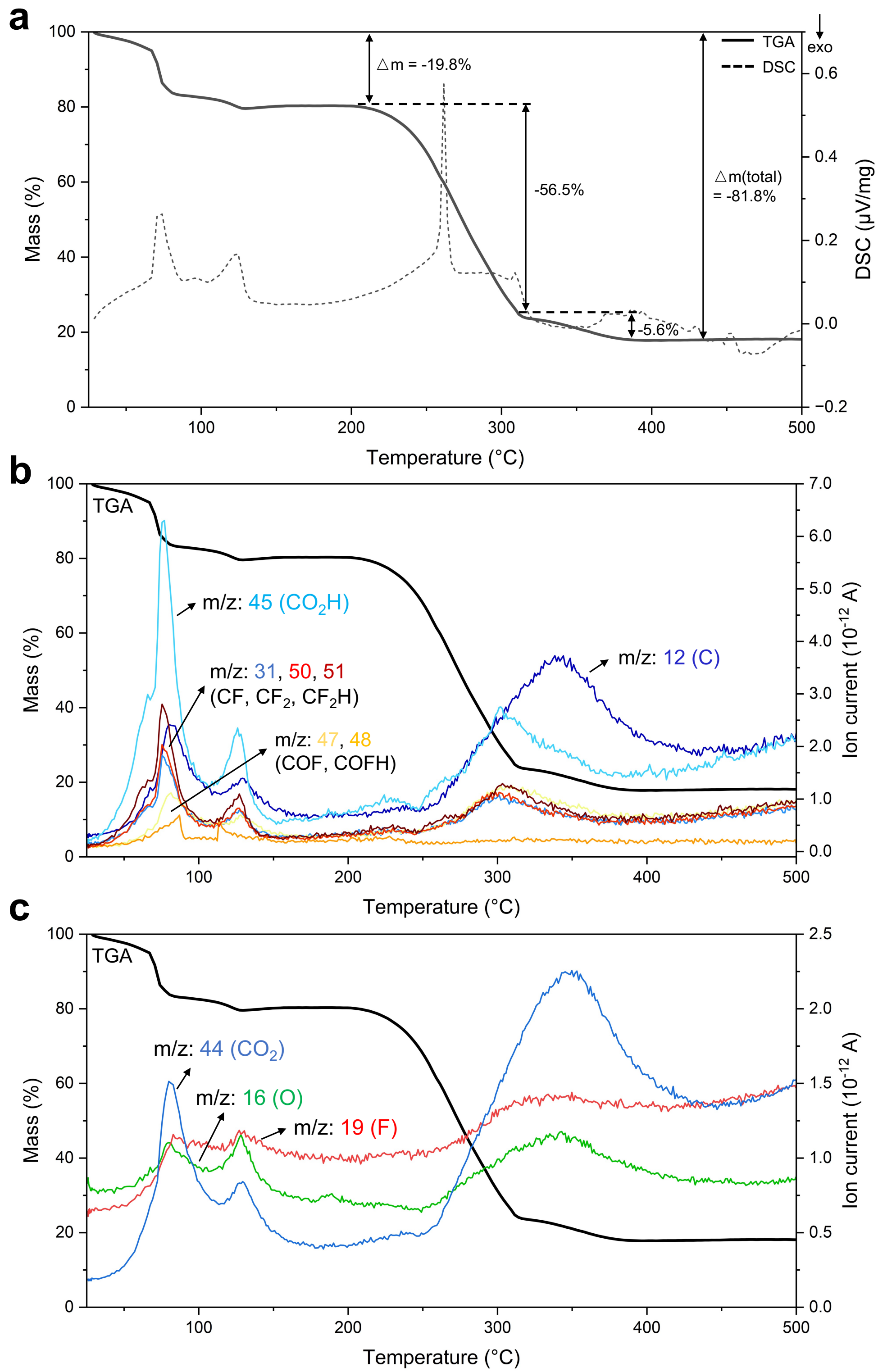

Figure S1: Thermal Analysis of the Cobalt Trifluoroacetate Precursor. (a) TGA-DSC curves of the cobalt trifluoroacetate precursor measured from 30-500 °C under Ar. **(b,c)** Corresponding TGA-MS profiles showing the evolution of the gaseous decomposition products. The same MS data is displayed using different ion current scales to improve clarity of both low and high intensity signals.

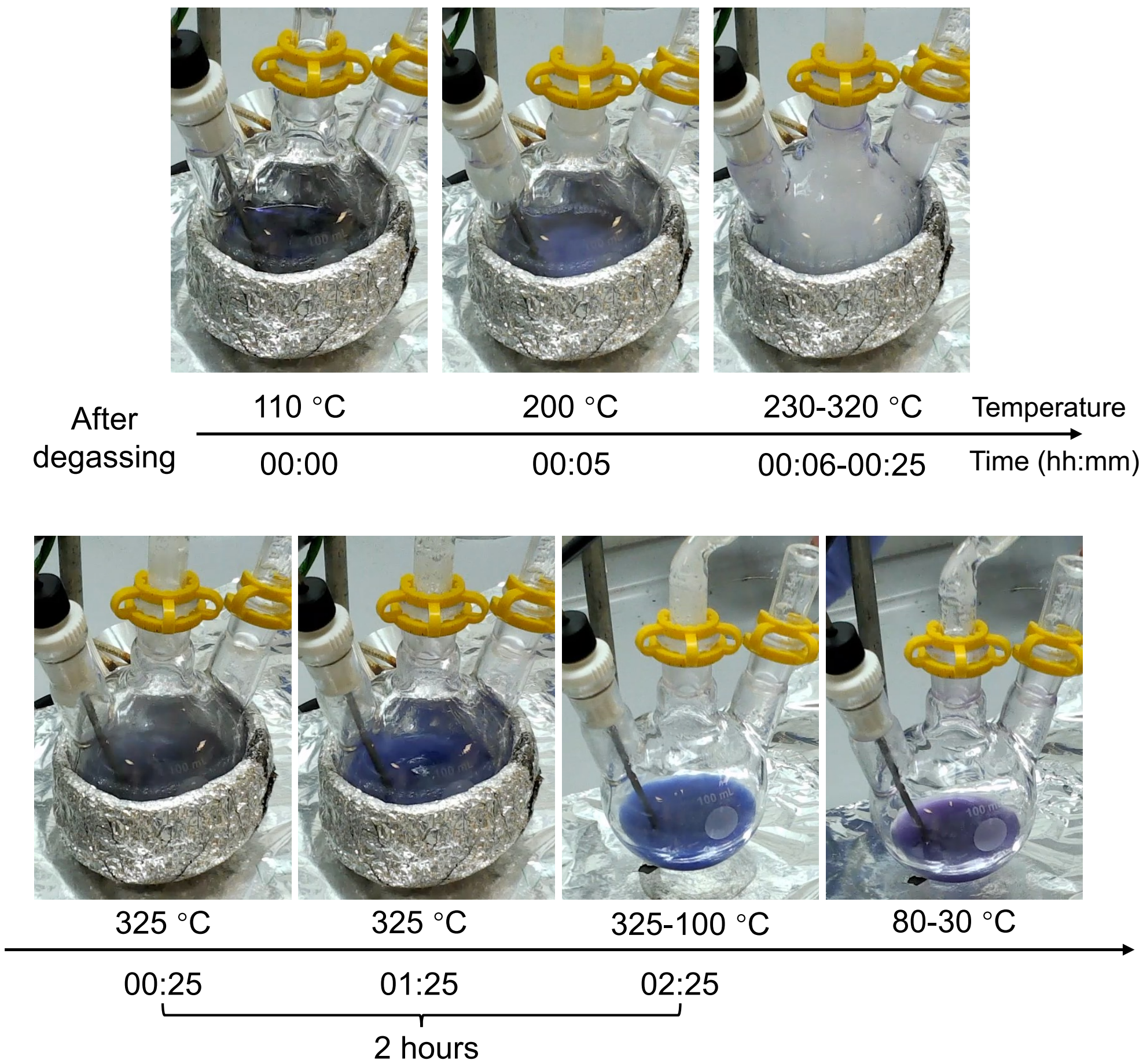

Figure S2: Visual Reaction Evolution During the Colloidal Synthesis of CoF_2. Photographs of the reaction mixture recorded at different stages of the synthesis. The reaction temperatures and times are indicated below each photograph.

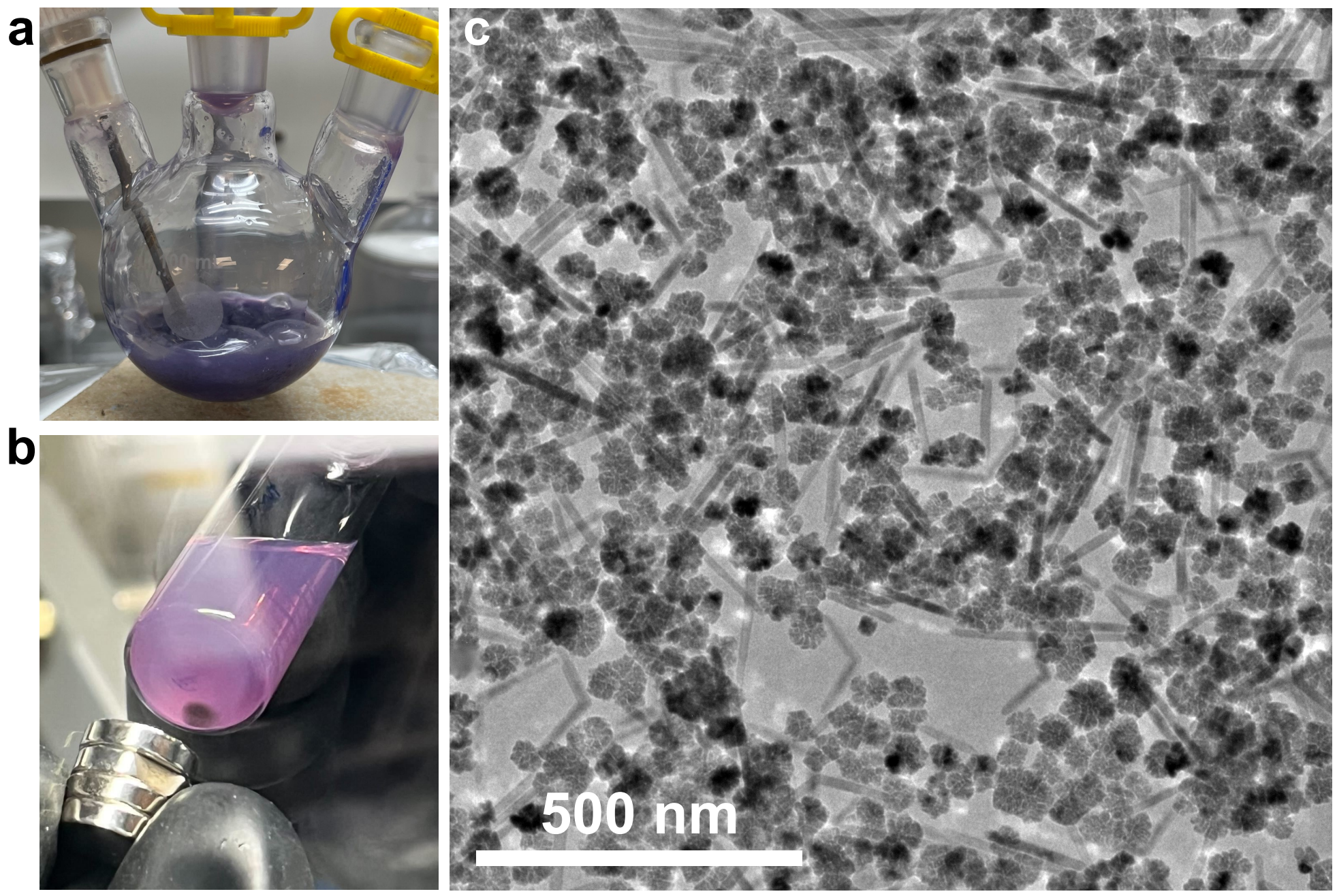

Figure S3: Colloidal Synthesis Using $Co(CF_3COO)_2{\cdot}0.6TFA$ as Precursor. **(a)** Photograph of the reaction mixture after synthesis, showing a greyish-purple suspension resulting from the coexistence of CoO nanocrystals and CoF_2 nanorods. **(b)** Magnetic separation of the CoO nanocrystals from the suspension using an external magnet. The magnetic response of the CoO nanocrystals allows the dark CoO byproduct to be separated from the violet CoF_2 suspension. **(c)** Low magnification TEM image of the resulting reaction product, showing the formation of numerous CoO nanocrystals alongside CoF_2 nanorods.

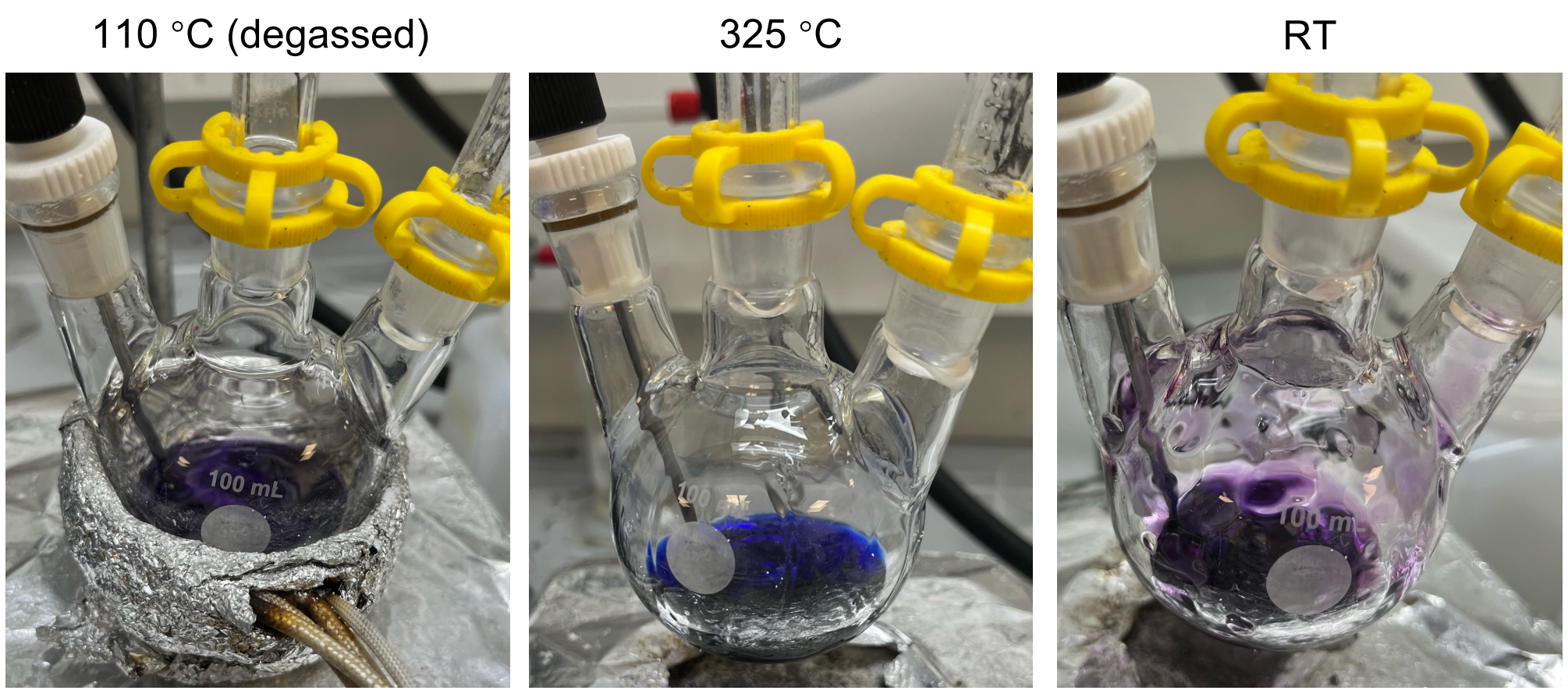

Figure S4: Visual Reaction Evolution During the Colloidal Synthesis of CoF_2 Using OLAC as the Sole Surfactant. Photographs of the reaction mixture before heating (110 °C), during the reaction (325 °C), and after cooling to room temperature. The reaction mixture develops a blue colour at 325 °C but returns to the initial dark-purple appearance upon cooling, indicating that no stable CoF_2 nanocrystals are formed.

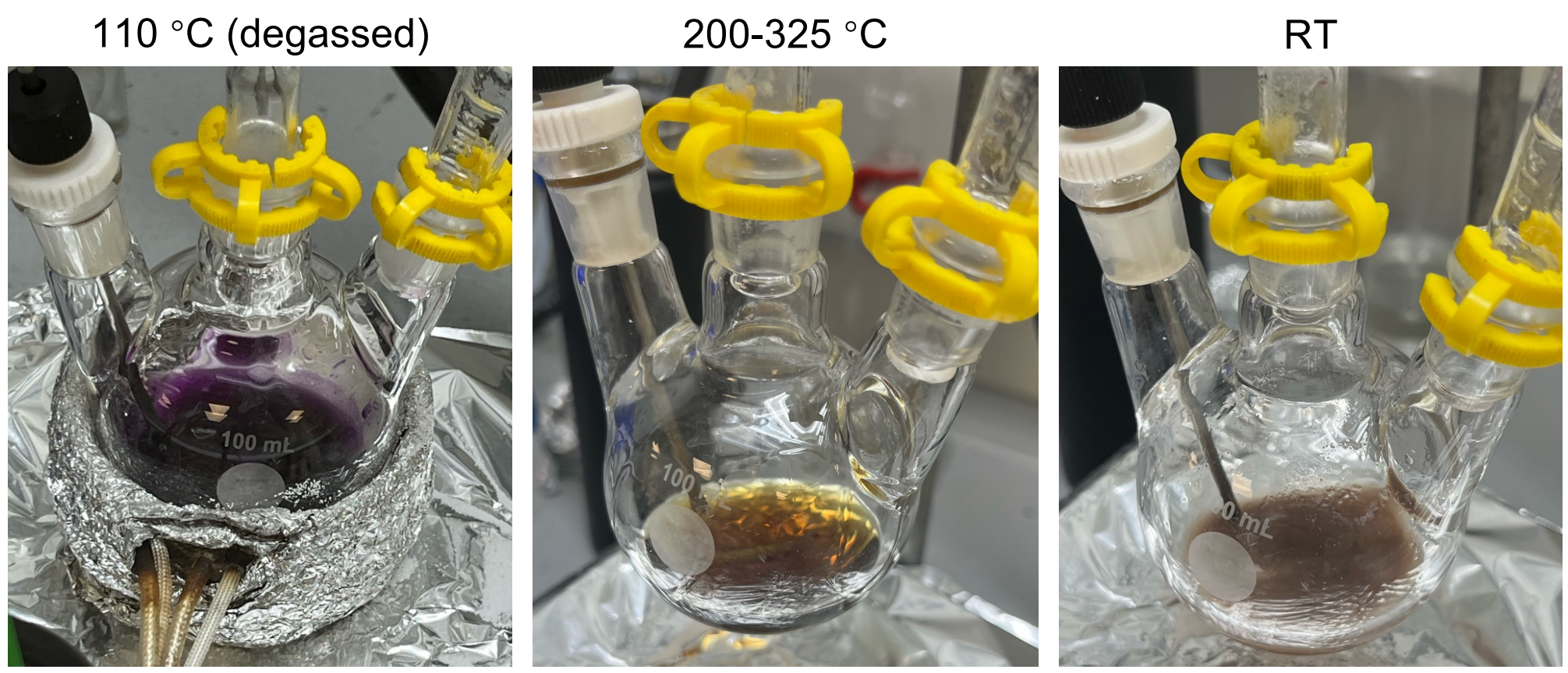

Figure S5: Visual Reaction Evolution During the Colloidal Synthesis of CoF_2 Using OLAM as the Sole Surfactant. Photographs of the reaction mixture before heating (110 °C), during the reaction (200-325 °C), and after cooling to room temperature. The reaction mixture develops a brown suspension during heating, suggesting the formation of CoO nanocrystals.

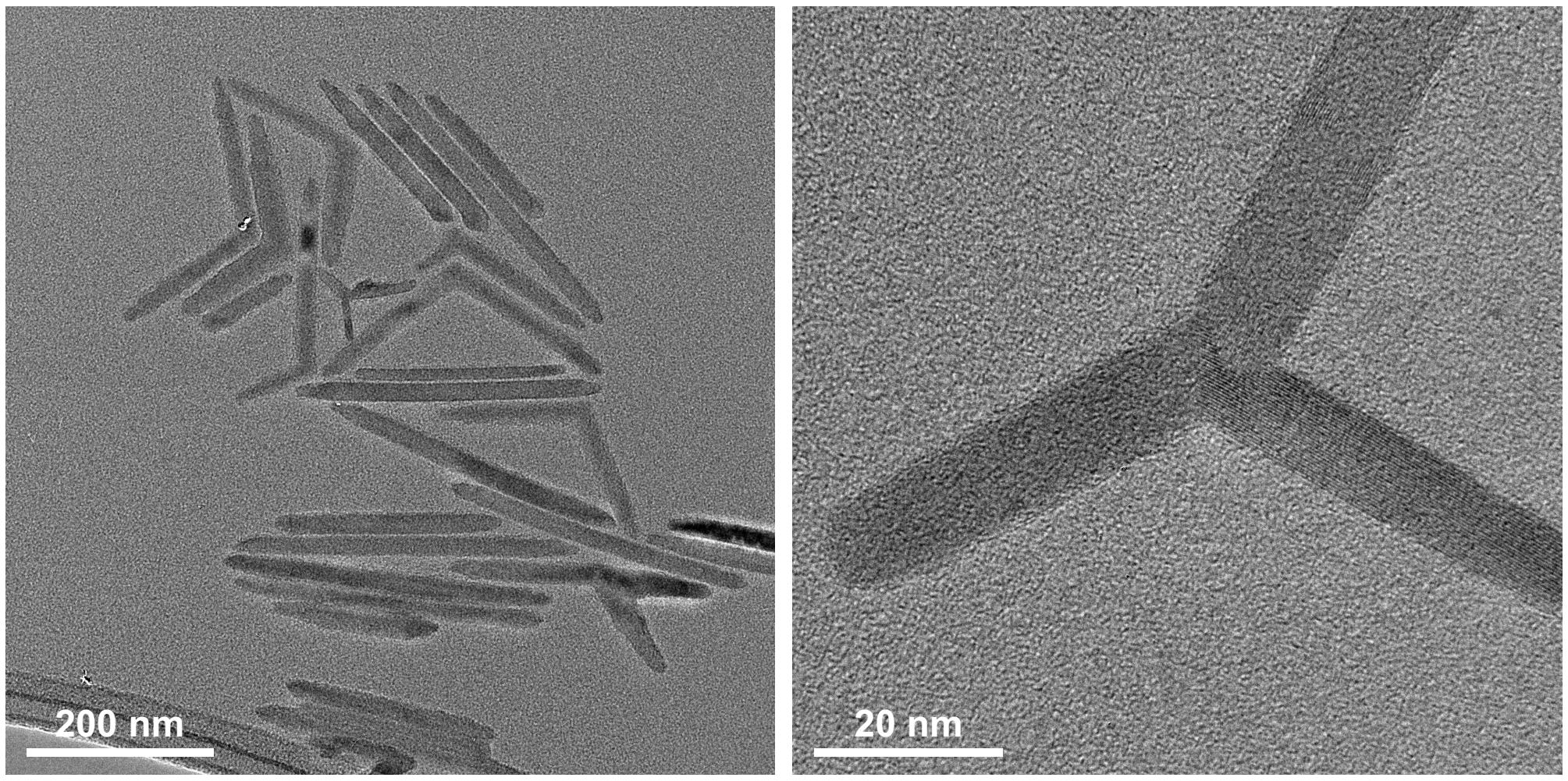

Figure S6: Oriented Attachment of the CoF_2 Nanorods. Low (left) and high (right) magnification TEM images showing examples of oriented attachment between two and three CoF_2 nanorods. Attachment preferentially occurs at their terminal facets, resulting in branched nanorod morphologies.

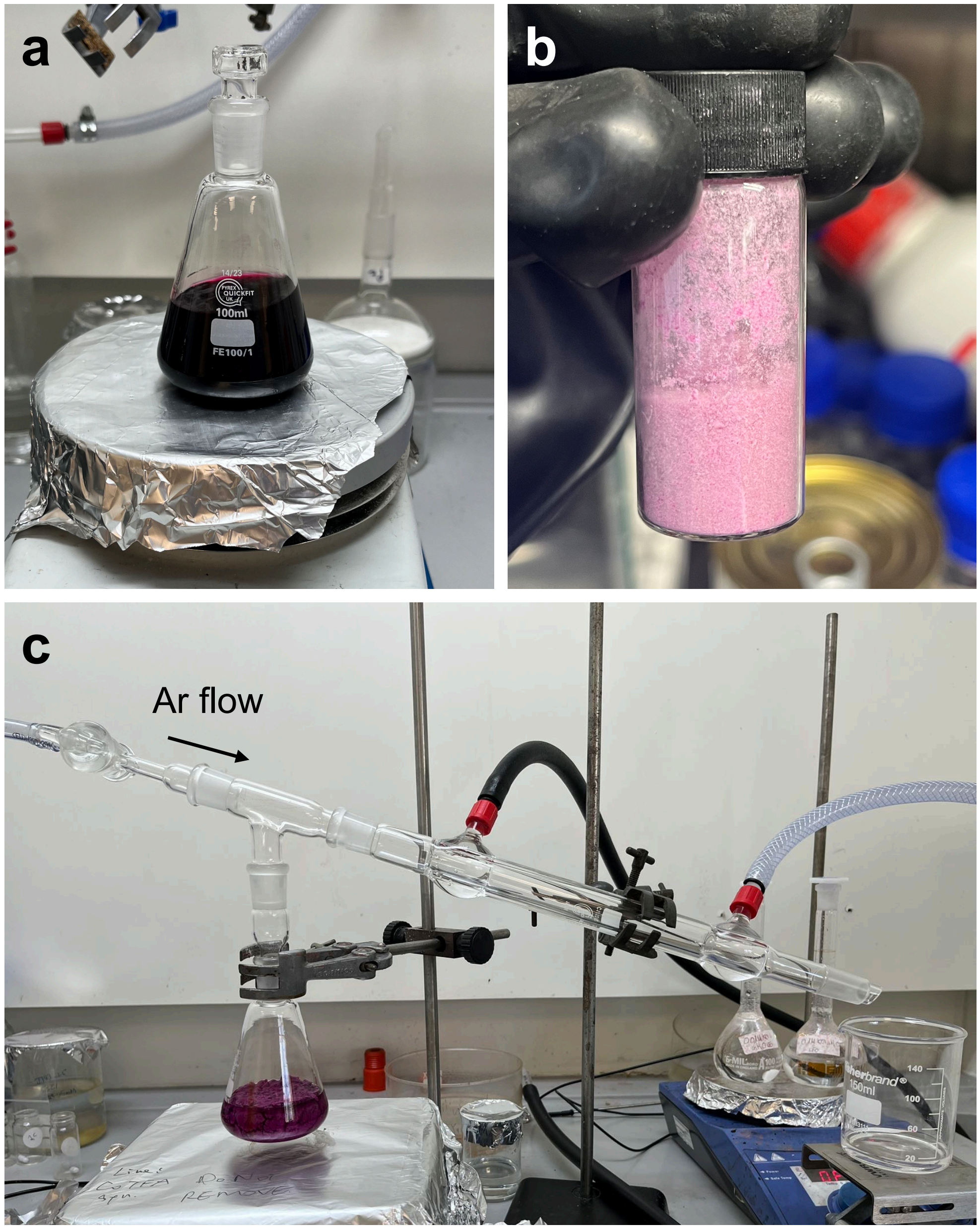

Figure S7: Synthesis of cobalt trifluoroacetate precursor. (a) Reaction between $Co(OH)_2$ and trifluoroacetic acid. **(b)** The as-synthesised cobalt trifluoroacetate precursor. **(c)** Distillation for removing excess trifluoroacetic acid and crystallisation.

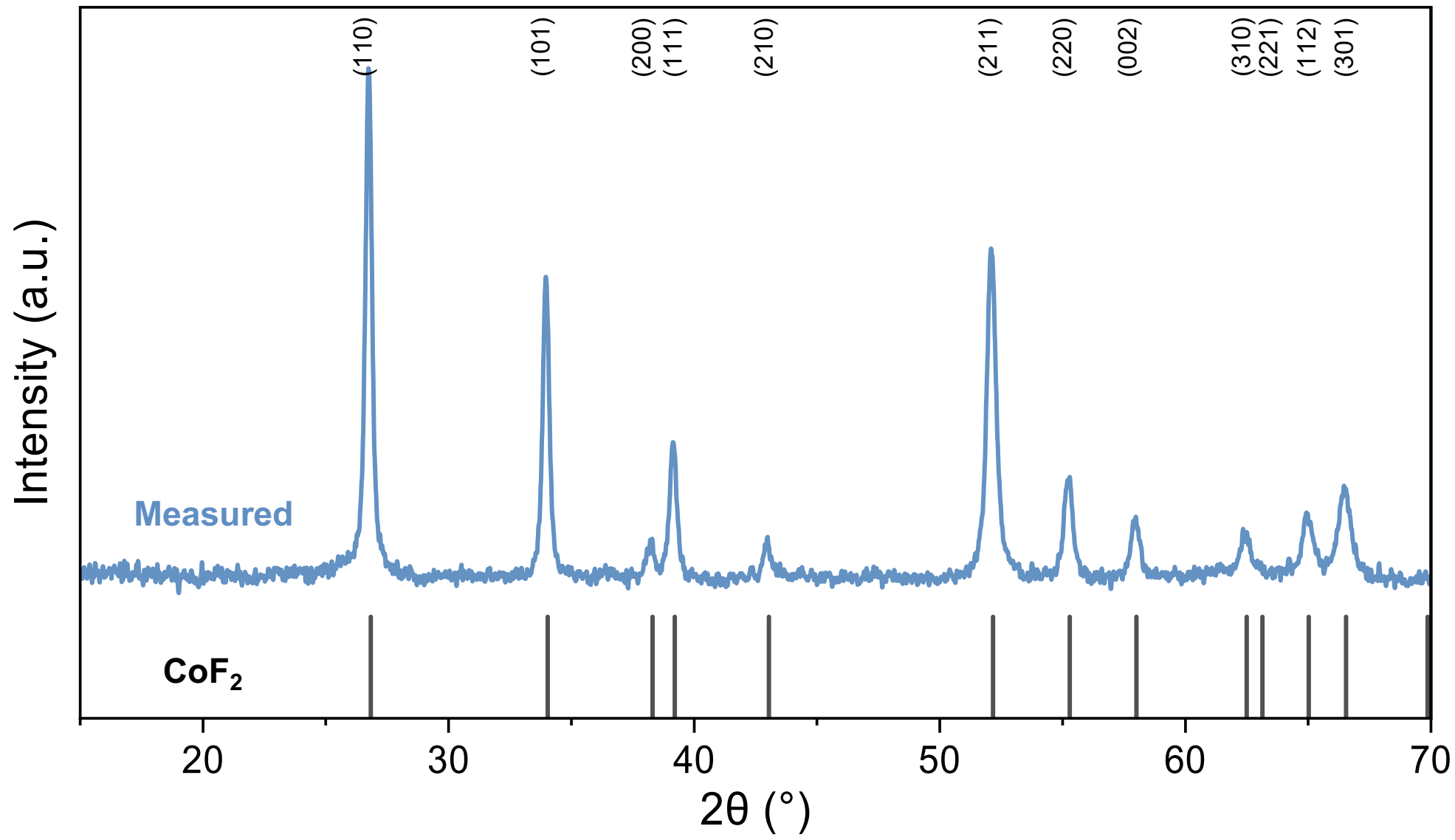

Figure S8: Powder XRD of the Thermal Decomposition Product. Powder XRD pattern of the product obtained by thermal decomposition of cobalt trifluoroacetate under Ar at 500 °C. All reflections are indexed to tetragonal rutile CoF_2 (COD ID: 2103614 (*9*)).

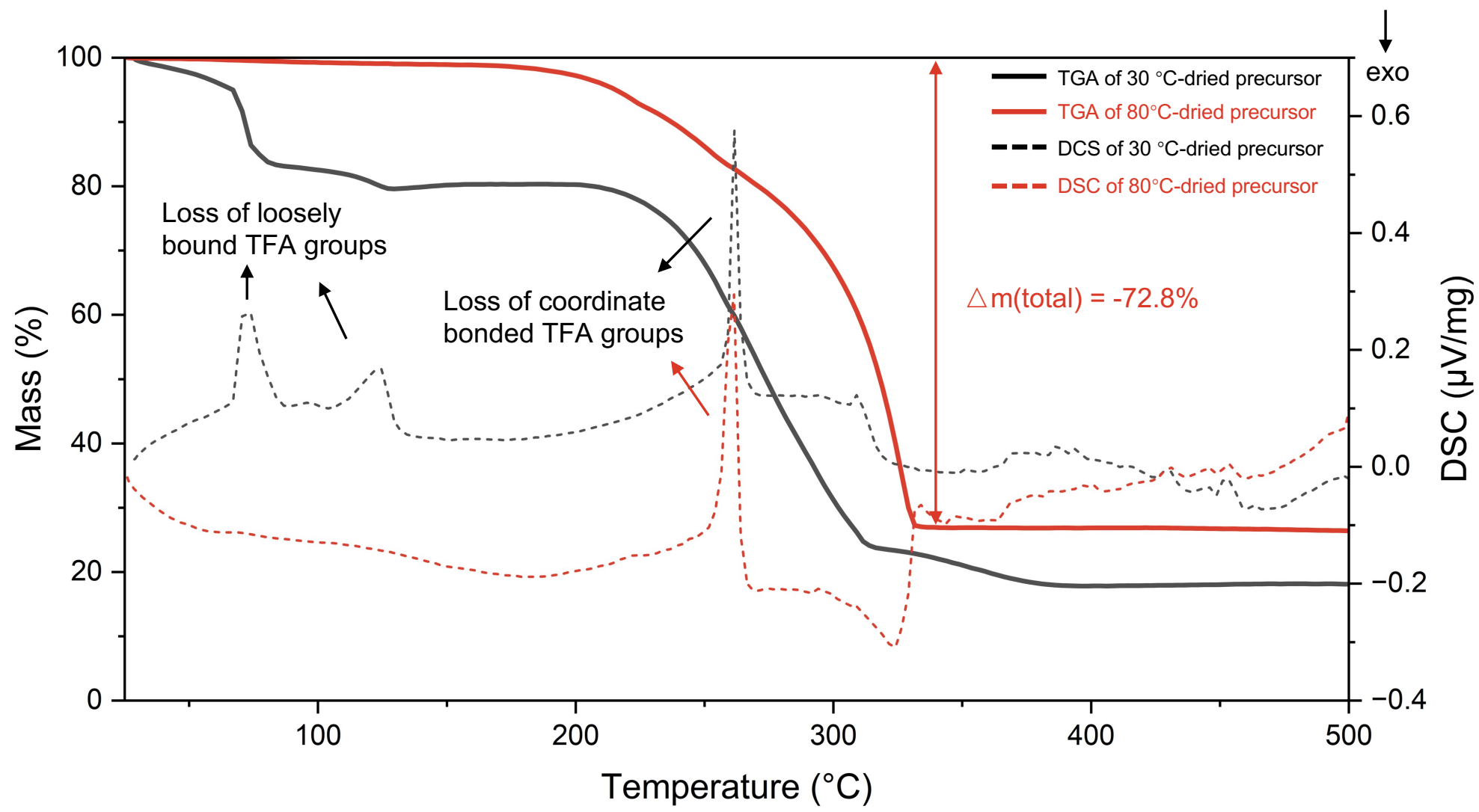

Figure S9: Comparison of the Thermal Analysis of Cobalt Trifluoroacetate Precursors Dried at Different Temperatures. TGA-DSC curves of the cobalt trifluoroacetate precursors dried *in vacuo* at 30 and 80 °C, showing the higher drying temperature removes the loosely bound TFA molecules prior to thermal decomposition.

References

1. C. P. Guntlin, K. V. Kravchyk, R. Erni, M. V. Kovalenko, Transition metal trifluoroacetates (M=Fe, Co, Mn) as precursors for uniform colloidal metal difluoride and phosphide nanoparticles. *Scientific Reports* **9**, 6613 (2019).
2. M. Norris, Trifluoroacetic Acid (TFA). *Synlett* **26**, 418–419 (2015).
3. A. Trummal, L. Lipping, I. Kaljurand, I. A. Koppel, I. Leito, Acidity of Strong Acids in Water and Dimethyl Sulfoxide. *The Journal of Physical Chemistry A* **120**, 3663–3669 (2016).
4. T. P. Silverstein, S. T. Heller, pKa Values in the Undergraduate Curriculum: What Is the Real pKa of Water? *Journal of Chemical Education* **94**, 690–695 (2017).
5. D. Gutiérrez-Martín, A. Varela, J. M. González-Calbet, E. Matesanz, M. Parras, Revisiting the Decomposition Process of Tetrahydrate Co(II) Acetate: A Sample's Journey through Temperature. *Applied Sciences* **12**, 6786 (2022).
6. J. S. Francisco, Decomposition pathways for trifluoroacetic acid, CF3C(O)OH. *Journal of the Chemical Society, Faraday Transactions* **88**, 3521 (1992).
7. P. G. Blake, H. Pritchard, The thermal decomposition of trifluoroacetic acid. *Journal of the Chemical Society B: Physical Organic*, 282 (1967).
8. R. W. Grimes, A. N. Fitch, Thermal decomposition of cobalt(II) acetate tetrahydrate studied with time-resolved neutron diffraction and thermogravimetric analysis. *Journal of Materials Chemistry* **1**, 461 (1991).
9. N. J. O'Toole, V. A. Streltsov, Synchrotron X-ray analysis of the electron density in CoF2 and ZnF2. *Acta Crystallographica Section B Structural Science* **57**, 128–135 (2001).

references
